# Room-temperature multiple ligands-tailored SnO_2_ quantum dots endow in situ dual-interface binding for upscaling efficient perovskite photovoltaics with high *V*_OC_

**DOI:** 10.1038/s41377-021-00676-6

**Published:** 2021-12-02

**Authors:** Zhiwei Ren, Kuan Liu, Hanlin Hu, Xuyun Guo, Yajun Gao, Patrick W. K. Fong, Qiong Liang, Hua Tang, Jiaming Huang, Hengkai Zhang, Minchao Qin, Li Cui, Hrisheekesh Thachoth Chandran, Dong Shen, Ming-Fai Lo, Annie Ng, Charles Surya, Minhua Shao, Chun-Sing Lee, Xinhui Lu, Frédéric Laquai, Ye Zhu, Gang Li

**Affiliations:** 1 Department of Electronic and Information Engineering, Research Institute for Smart Energy (RISE), Guangdong-Hong Kong-Macao (GHM) Joint Laboratory for Photonic-Thermal-Electrical Energy Materials and Devices, The Hong Kong Polytechnic University, Hung Hom, Kowloon, Hong Kong China; 2 Department of Electrical and Computer Engineering, Nazarbayev University, Nur-Sultan, Kazakhstan; 3 The Hong Kong Polytechnic University Shenzhen Research Institute, Shenzhen, 518057 China; 4 Hoffmann Institute of Advanced Materials, Shenzhen Polytechnic, 7098 Liuxian Boulevard, Shenzhen, 518055 China; 5 Department of Applied Physics, The Hong Kong Polytechnic University, Hung Hom, Kowloon, Hong Kong China; 6 Department of Chemical and Biological Engineering, The Hong Kong University of Science and Technology, Clear Water Bay, Kowloon, Hong Kong China; 7 King Abdullah University of Science and Technology (KAUST), KAUST Solar Center (KSC), Physical Sciences and Engineering Division (PSE), Material Science and Engineering Program (MSE), Thuwal, 23955-6900 Kingdom of Saudi Arabia; 8 Department of Physics, The Chinese University of Hong Kong, Shatin, 999077 Hong Kong China; 9 Center of Super-Diamond and Advanced Films (COSDAF), Department of Chemistry, City University of Hong Kong, Hong Kong, China

**Keywords:** Electronics, photonics and device physics, Optics and photonics

## Abstract

The benchmark tin oxide (SnO_2_) electron transporting layers (ETLs) have enabled remarkable progress in planar perovskite solar cell (PSCs). However, the energy loss is still a challenge due to the lack of “hidden interface” control. We report a novel ligand-tailored ultrafine SnO_2_ quantum dots (QDs) *via* a facile rapid room temperature synthesis. Importantly, the ligand-tailored SnO_2_ QDs ETL with multi-functional terminal groups in situ refines the buried interfaces with both the perovskite and transparent electrode *via* enhanced interface binding and perovskite passivation. These novel ETLs induce synergistic effects of physical and chemical interfacial modulation and preferred perovskite crystallization-directing, delivering reduced interface defects, suppressed non-radiative recombination and elongated charge carrier lifetime. Power conversion efficiency (PCE) of 23.02% (0.04 cm^2^) and 21.6% (0.98 cm^2^, *V*_OC_ loss: 0.336 V) have been achieved for the blade-coated PSCs (1.54 eV *E*_g_) with our new ETLs, representing a record for SnO_2_ based blade-coated PSCs. Moreover, a substantially enhanced PCE (*V*_*OC*_) from 20.4% (1.15 V) to 22.8% (1.24 V, 90 mV higher *V*_*OC*_, 0.04 cm^2^ device) in the blade-coated 1.61 eV PSCs system, via replacing the benchmark commercial colloidal SnO_2_ with our new ETLs.

## Abstract (web summary)

The ligand-tailored SnO_2_ QDs ETL with multi-functional terminal groups in situ refines the buried interfaces with both the perovskite and transparent electrode via enhanced interface binding and perovskite passivation.

## Graphical abstract

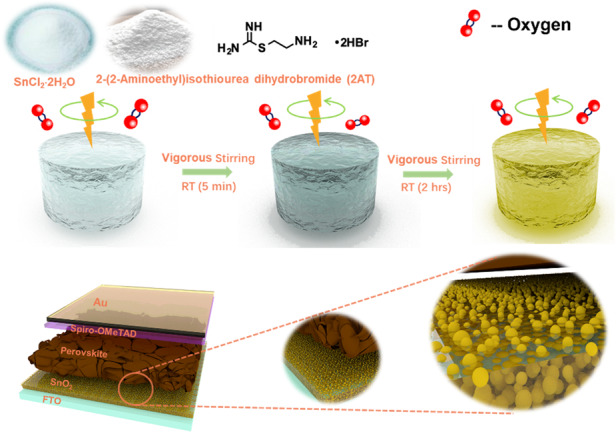

## Introduction

The organic–inorganic hybrid perovskite (OIHP) materials have attracted enormous research interests^1–6^, owing to the unique merits including material abundancy, low cost, long carrier diffusion lengths up to micrometer scale^7^, tunable bandgap^8^, high defects tolerance^4^, outstanding and bipolar carrier transport properties^9,10^, and high absorption coefficient^11,12^. OIHPs have been highlighted to be promising candidates for various optoelectronic applications, such as lasers^13^, photodetectors^14^, radiation detectors^15^, memories^16^, water splitting, X-ray imagers^17,18^, light emission diodes (PeLEDs)^19^, and in particular solar cells. At present, mesoporous titanium oxide (TiO_2_) nanostructures are the efficient electron transporting layer (ETL) materials for regular n–i–p structure PSCs, delivering a certified PCE of 25.2% with stable power output^20^. Although mesoporous TiO_2_ ETL-based PSCs lead the efficiency competition, the high-crystallization annealing temperature is required to ensure high carrier mobility and high film quality of TiO_2_, which leads to longer energy payback time, also hinders the application in flexible and stretchable devices. Moreover, TiO_2_-based planar PSCs tend to suffer more photocatalytic issues^21^.

It is expected that a reliable ETL for efficient PSCs should possess good energy alignment, high mobility, good stability, and high transmittance^22^. Recently, SnO_2_-based ETLs^1,6,23–26^ have been extensively utilized in PSCs and are considered as the most promising candidate to replace TiO_2_. SnO_2_ owns a wide bandgap (*E*_g_) above 3.8 eV and favorable conduction band edge facilitating more efficient charge transfer^27^. Furthermore, SnO_2_ exhibited outstanding chemical stability, less photocatalytic degradation, and good electron mobility^22,28^. Intense research efforts have targeted on the development of highly efficient SnO_2_ ETLs that allow PCEs to boost up to 23.3% based on commercial colloidal SnO_2_ ETL, with a low hysteresis, valued by h-index ((PCE_reverse_−PCE_forward_)/PCE_reverse_)^1,6,23–25,29–35^. Although positive progresses have been demonstrated in those SnO_2_-based PSCs, the electronic and structural properties (e.g., conductivity, physical and electrical contact, work function, defects level) of SnO_2_ still highly depend on the synthesis approaches and fabrication conditions. On the other hand, polycrystalline PSCs inevitably possess a large number of crystallographic defects^36–38^, such as interfacial dangling bonds and uncoordinated ions (I^−^, Pb^2+^, Cs^+^, Rb^+^, K^+^), which result in increased nonradiative recombination and potential hysteresis issue. Hence, modifications or post-treatments (e.g., metallic ion doping, functional molecular coordinating, semiconducting polymers coating, fullerenes, and fullerene derivatives anchoring) of SnO_2_ ETLs have been introduced to alleviate interfacial charge recombination and promote carrier extraction, delivering favorable electronic and physical interfacial contact^39–44^. Unfortunately, most of these reported approaches incorporate the additional modification layer which inevitably increases the uncertainty (e.g., complexity, cost, and unreliability) during the scalable manufacturing procedure of PSCs. Moreover, weak interactions between the additional modifier(s) and ETL could be an issue on interfacial electronic contact and device stability^45^. In addition, reinforced interfacial interactions and high-quality low-temperature processed SnO_2_ ETLs are critical for flexible and stretchable PSC device manufacturing.

In this work, we report a facile, ligands-assisted, rapid formulated room-temperature synthetic approach for novel multi-functional terminal groups anchored SnO_2_ QDs, which function as excellent ETLs to in situ manipulate the interfacial contact in planar perovskite solar cells. Such ligand-tailored SnO_2_ QDs exhibited superior properties over the current benchmark materials (e.g., alcohol-based SnO_2_ and commercialized colloidal SnO_2_). The ligand-tailored SnO_2_ QDs ETLs in planar PSCs show threefold benefits: 1. the ligand-tailored SnO_2_ QDs not only passivated perovskite at the buried interface with ETL, but also work as a seeding-controlling agent to direct the subsequent high-quality perovskite crystallization, resulting in suppressed non-radiative recombination and elongated charge carrier lifetime; 2. the ultrafine SnO_2_ QDs act as interfacial “glue” to bridge the perovskite and transparent electrode both chemically and physically, resulting in a favorable electronic and physical interfacial contact; 3. The new SnO_2_ QDs ETLs allow the crystallization temperature to be lowered to 100 °C, providing a new opportunity for flexible substrate manufacturing. The substantial suppressed non-radiative recombination and reduced buried interface defects are supported with enhanced electroluminescence quantum efficiency (ELQE). In addition, the device stability (humidity, thermal, UV) is noticeably improved. With these improved aspects, the ligand-tailored SnO_2_ QDs ETL-based devices achieve a high PCE (reverse scan (RS)) of 23.02% (22.51% verified by an ISO/IEC 17025:2005 accredited certification laboratory) in a 1.541 eV bandgap perovskite system. It is noteworthy that a substantially enhanced PCE (*V*_OC_) from 20.4% (1.152 V) to 22.8% (1.242 V, 90 mV higher *V*_OC_, 0.04 cm^2^ device) in the blade-coated 1.613 eV PSCs system, via replacing the benchmark commercial colloidal SnO_2_ ETL with our ligand-tailored SnO_2_ QDs. We further investigate the feasibility of the ultrafine SnO_2_ QDs ETL in the upscaling of different PSC systems (*E*_g_ = 1.541 and 1.613 eV, respectively, Fig. S1) via manufacturing friendly blade-coating process. We achieve blade-coated devices with 21.6% (0.98 cm^2^, an impressive *V*_OC_ loss of only 0.336 V) in a 1.541 eV and 20.7% PCE (0.8 cm^2^) in a 1.613 eV perovskite system, representing a record PCE for SnO_2_ ETL-based upscaling blade-coated PSCs to the best of our knowledge. Moreover, we successfully achieved 30 × 30 mm^2^ mini-modules with 19.5% PCE in 1.541 eV (two sub-cells) and 18.9% in 1.613 eV (three sub-cells) perovskite systems. Therefore, the in situ solution chemistry engineering of metal oxide synthesis contrives a new direction toward achieving low temperature and high-quality functionalized SnO_2_ ETLs, and is compatible with upscaling of large-area high-quality films in perovskite-based photoelectronic devices.

## Results

**Fig. 1:**
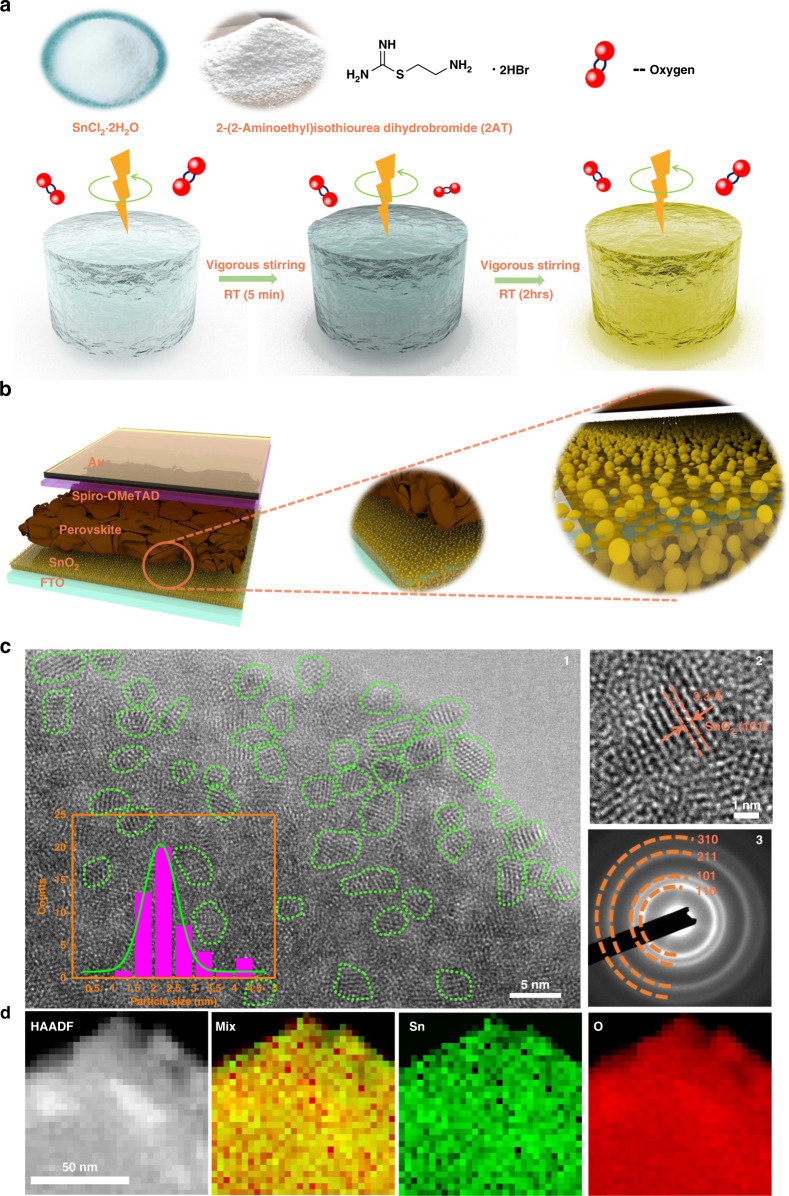
Synthesis method and morphology of the ligand-tailored SnO_2_ QDs. **a** Schematic of a facile one-step aqueous wet-chemical approach to rapid formulate multi-functional SnO_2_ QDs. **b** Schematic of a planar perovskite solar cell device structure using an electron transporting layer of SnO_2_ QDs. **c** 1. TEM image of SnO_2_ QDs coated on a carbon support copper grid. Inset shows the particle size distribution of SnO_2_ QDs. Note that the irregular green circle is the guideline of the SnO_2_ QDs edge. 2. High-resolution TEM of SnO_2_ QDs. The lattice fringes of the (110) crystal plane with a space distance of around 0.33 nm are clearly observed. 3. Selected area electron diffraction (SAED) pattern of SnO_2_ QDs. Electron reflection rings from the (110), (101), (211), and (310) crystal planes of SnO_2_ were clearly observed. **d** Selected area of high-angle annular dark-field (HAADF) STEM image of SnO_2_ QDs and the corresponding STEM-EELS maps of Sn and O signals

The organic ligand 2-(2-aminoethyl) isothiourea dihydrobromide (2AT) was introduced in SnO_2_ QDs synthesis, on the basis that it possesses two types of organic amine terminal groups (a primary amine and a formamidine), which not only facilitate the stabilization and in situ multi-functionalization of the SnO_2_ QDs but also allow for favorable interfacial passivation through chemical bonding. The chemical structure of the 2AT additive is shown in Fig. 1a, which contains multiple functional terminal groups, including amino (–NH_2_), imino (–NH), 2-aminoethyl (–CH_2_–CH_2_–NH_2_), and bromide (–Br). The SnO_2_ QDs solution was synthesized by hydrolysis of tin source (SnCl_2_·2H_2_O) with the assist of a small amount of 2AT additive under O_2_-rich ambient at room temperature via vigorous stirring. Generally, tin source (SnCl_2_·2H_2_O) experiences the following main reaction processes (hydrolysis, dehydration, and oxidation)^46^:

1 $${{{\mathrm{SnCl}}}}_2 + 2{{{\mathrm{H}}}}_2{{{\mathrm{O}}}} \to {{{\mathrm{Sn}}}}\,\left( {{{{\mathrm{OH}}}}} \right)_2 + 2{{{\mathrm{HCl}}}}\,\left( {{{{\mathrm{hydrolysis}}}}} \right)$$

2 $${{{\mathrm{Sn}}}}\,\left( {{{{\mathrm{OH}}}}} \right)_2 + {{{\mathrm{O}}}}_2 \to {{{\mathrm{SnO}}}}_2 + {{{\mathrm{H}}}}_2{{{\mathrm{O}}}}\left( {{{{\mathrm{dehydration}}}}\,{{{\mathrm{and}}}}\,{{{\mathrm{oxidation}}}}} \right)$$

During the hydrolysis step, white precipitate, Sn(OH)Cl, Sn(OH)_2_, can be generated once SnCl_2_·2H_2_O was dissolved into deionized water due to its rapid hydrolysis nature, and then undergoes a dehydration-oxidation process^46,47^. Consequently, SnO_2_ QDs transparent yellow aqueous solution can be obtained. Previous studies have demonstrated that SnCl_2_·2H_2_O could gradually self-convert into SnO_2_ colloids via a hydrolysis-oxidation process. However, the resultant aqueous solution is extremely unstable in which SnO_2_ colloids are prone to aggregate and precipitate, and the synthesis process is time-consuming (several days). In our new solution chemistry strategy, with the assist of 2AT additive, the milky suspension rapidly transforms into a semitransparent bright SnO_2_ solution and then converts into the yellowish SnO_2_ solution within 2 h (Figs. 1a and S2), which can be stored for over 3 months without any aggregation and precipitation. These results indicated that the new 2AT additive not only helps in accelerating the hydrolysis–dehydration–oxidation process of SnCl_2_·2H_2_O, but also renders the resultant SnO_2_ solution immune to aggregation and precipitation so that good chemical stability can be attained. This is because the consumption of the hydrogen chloride (see Eq. (1)) by the presence of 2AT additive largely enables an ongoing hydrolysis process and finally accelerated the formation of SnO_2_ QDs solution. Moreover, thanks to the multi-functional terminal groups of 2AT additive, metal ion (Sn^2+^, Sn^4+^) could be chemically interacted with S functionalities of 2AT while the protonated organic amine terminal groups with positive charges are surrounding the as-prepared SnO_2_ QDs^36,46,48^, delivering small particle size and suppressed solution aggregation. In addition, these functional groups possibly facilitate the formation of more nuclei at the beginning of the hydrolysis process and thus smaller SnO_2_ particle size can be obtained due to suppressed segregation by the protonated functional groups. Similar colloidal QDs size control phenomena have been reported in copper indium sulfide/zinc sulfide (CIS/ZnS) core/shell system, cadmium selenide (CdSe) QDs, etc.^31,49,50^.

To check the difference between our SnO_2_ QDs and other SnO_2_, we performed the dynamic light scattering (DLS) measurement as shown in Fig. S3. In the commercial SnO_2_ NPs solution, DLS showed three Gaussian distributions—i.e., three different sizes (~2, ~13, and ~2500 nm), which is in good agreement with the previous literatures^32,51^. The DLS of the c-SnO_2_ also shows three Gaussian peaks (~1, ~5, ~150 nm). While in the 2AT–SnO_2_ QDs solution, we observed only one Gaussian distribution in small size (~7 nm), which indicated that 2AT–SnO_2_ QDs are uniformly distributed in the solution with a small deviation in particle size. It should be noted that the method of DLS usually overestimates the absolute particle or cluster size due to the multiple scattering process and electrical double layer around the particle^51,52^. Furthermore, we investigate the influence of 2AT concentration on SnO_2_ particle size (Fig. S4). During the synthesis process, in the case of optimal 2AT concentration (also further increasing concentration of 2AT by 5 times), DLS shows only one Gaussian distribution in small size (~7 nm). If too less 2AT (e.g., by decreasing the concentration of 2AT by 10 times during the synthesis process), we observed two Gaussian distributions in small size (~5 nm) and large size (~150 nm). This suggests that insufficient 2AT leads to the partial segregation of SnO_2_ QDs.

**Fig. 2:**
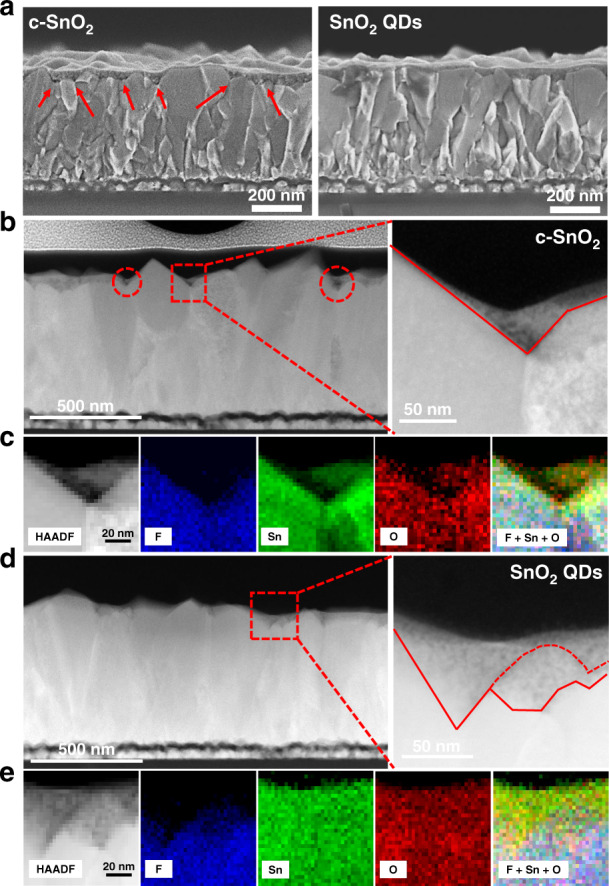
Cross-sectional morphology of ETL coated FTO. **a** SEM cross-sectional image of c-SnO_2_-coated FTO (left) and SnO_2_ QDs-coated FTO (right). HAADF-STEM cross-sectional image (left) and zoom-in image of the selected area (right) of **b** c-SnO_2_-coated FTO and **c** SnO_2_ QDs-coated FTO. HAADE-STEM-EELS cross-sectional element mapping of **d** c-SnO_2_-coated FTO and **e** SnO_2_ QDs-coated FTO

We incorporated SnO_2_ QDs equipped with multi-functional groups as ETL in planar PSCs. SnO_2_ QDs-based planar PSC with the architecture of fluorine-doped SnO_2_ (FTO)/SnO_2_ QDs/perovskite/spiro-OMeTAD/Au, as demonstrated in Figs. 1b and S5. The morphology of SnO_2_ QDs was confirmed by transmission electron microscopy (TEM). Figure 1c (1) and Fig. S6 shows the SnO_2_ QDs with an average size of 2–2.5 nm were uniformly distributed on the TEM copper grid. High-resolution TEM (HR-TEM) results suggested that the SnO_2_ QDs are highly crystallized which is beneficial to yield a defect-reduced SnO_2_ film. The lattice fringes of the (110) crystal plane with a space distance of around 0.33 nm are clearly observed as shown in Fig. 1c (2). According to the corresponding selected area electron diffraction (SAED) pattern in Fig. 1c (3), electron reflection rings from the (110), (101), (211), and (310) crystal planes of SnO_2_ were observed which suggest that the SnO_2_ QDs are polycrystalline. Scanning transmission electron microscopy (STEM) together with electron energy loss spectra (EELS) was performed to further characterize the SnO_2_ QDs. Figure 1d shows the selected area of high-angle annular dark-field (HAADF) STEM image and the corresponding EELS maps of Sn and O signals, indicating that the prepared SnO_2_ QDs films are composed of Sn and O elements. Scanning electron microscopy (SEM) is used to examine the surface morphology of SnO_2_-coated FTO glass substrate. For comparison, we investigated three types of SnO_2_ ETLs: alcohol-based SnO_2_ (c-SnO_2_), commercialized SnO_2_ nanoparticles (NPs), and SnO_2_ QDs. The bare FTO substrate shows a clear and rough morphology due to the nature of large FTO grains (Fig. S7A). Among the three types of SnO_2_-coated FTO glass substrates, shown in Fig. S7B–D, the SnO_2_ QDs demonstrate a smoother and full coverage surface coating, evidenced by the less FTO features in the morphology by SEM (Fig. S7) and AFM images (Fig. S8). This agrees with the ultrafine size of the ligand-tailored SnO_2_ QDs which are beneficial for surface physical defects filling. Considering that the feature of FTO retains a rough surface (Fig. S7A), the quality of physical contact at the interface between the FTO and ETL should be a critical factor on obtaining high electron extraction efficiency with less interface defects. Recently, Choi and co-workers revealed that high surface roughness of FTO, surface energies mismatch, and volume shrinkage of ETL on FTO surface give rise to the formation of physical defects at the interfaces, led to a reduced physical contact area^53^. Similarly, Segawa group showed that a rough interface can trigger the local-heavy doping caused by the electrostatic dipole, resulting in tunneling phenomenon which can induce the hysteresis in the perovskite solar cell^54^. Based on the above reported experimental results, we performed SEM, STEM, and HR-TEM to explore the quality of interface contact between the FTO and ETL. In our study, we investigated the c-SnO_2_/FTO, SnO_2_ NPs/FTO, and SnO_2_ QDs/FTO to explore surface covering (or physical defects filling) ability of these ETLs on FTO substrate. We clearly observe a poor physical contact feature in the c-SnO_2_/FTO interface as shown in Fig. 2a, in which the red arrows reveal the distribution of physical defects. This suggests that the FTO grooves (or valleys) filling ability of c-SnO_2_ is much weaker than that of our SnO_2_ QDs (Fig. 2), and commercial SnO_2_ NPs (Fig. S9). To gain more solid evidence, focused ion beam (FIB) workstation equipped with an in situ micromanipulator was utilized to prepare samples for STEM and HR-TEM imaging. We prepared samples with the architecture of Pt/Carbon/ETL/FTO by lift-off FIB technique (see details in “Materials and methods” and Fig. S10A), as depicted in the HAADF-STEM cross-sectional images in Fig. 2b and d. We directly observed that the rough interface (ETL/FTO) is composed of many FTO nanopyramids. A significant difference in the physical contact of the FTO coated with c-SnO_2_ and SnO_2_ QDs (Fig. S10B and S10C) is noted. The dotted red circles (Fig. 2b) show examples of physical defects (the void area). In more detail, the observation on c-SnO_2_/FTO sample by HAADF-STEM image demonstrates that an uneven distribution feature of c-SnO_2_ (at areas that were very thick or very thin). This is mainly due to fact that alcoholysis processed c-SnO_2_ suffers from volume shrinkage of SnO_2_ films (for example, from SnCl_2_ to SnO_2_). Further enlarged HAADF-STEM image reveals that physical defects (the voids) at the valley of c-SnO_2_/FTO, which is further hammered by HAADE-STEM-EELS cross-sectional element mapping (Fig. 2c). As expected, all elements including F, Sn, and O are identified in c-SnO_2_/FTO, indicating that c-SnO_2_ is unevenly distributed (partial shortage, the void) at the protruding areas and the valley of FTO pyramids. As compared to the interface quality of the c-SnO_2_/FTO sample, the SnO_2_ QDs/FTO sample exhibit a highly uniform distribution of SnO_2_ QDs clinging to the FTO, resulting in a physical defect-free interface and reinforced interfacial contact as confirmed by further enlarged HAADF-STEM image (Fig. 2d). The above observations are much more convincingly proved by HAADE-STEM-EELS cross-sectional element mapping (Fig. 2e), indicating that the 2AT additive assisted synthesis process yields SnO_2_ QDs that fully cover the apexes and grooves (or valleys) of FTO pyramids, delivering a better interfacial physical contact between the FTO and ETL. This can be ascribed to the ultrafine quantum dot particle size of SnO_2_ crystallites, resulting in improved surface filling ability and suppressed volume shrinkage.

**Fig. 3:**
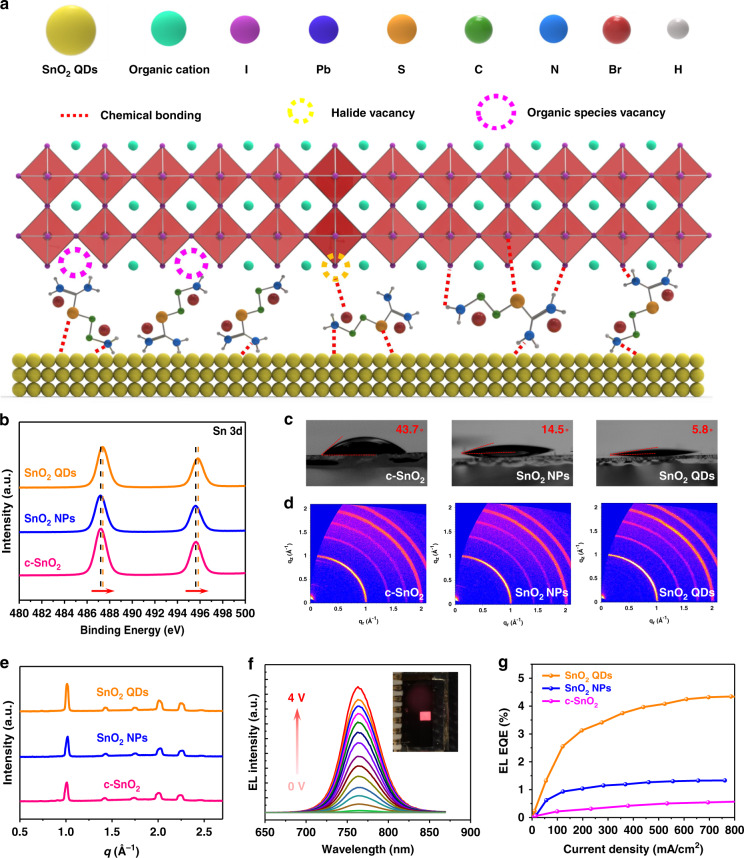
Passivation mechanism and spectroscopic characterization of perovskite films/devices with ligand-tailored SnO_2_ QDs. **a** Schematic illustration of proposed in situ interfacial defect passivation induced by utilizing ligand-tailored SnO_2_ QDs. **b** XPS core-level spectra of Sn 3*d* on c-SnO_2_, SnO_2_ NPs, and SnO_2_ QDs film. **c** The deionized water contact angles of various ETLs coated FTO substrates. **d** GIWAXS patterns of the perovskite films deposited on various ETLs coated quartz substrates. **e** The diffraction features of the corresponding intensity as a function of *q*. **f** EL emission spectra from SnO_2_ QD-based PSCs under different voltage bias. The inset displays a bright red emission (~770 nm) operating as a light-emitting diode (LED). **g** The plot of ELQE as a function of the injection current density

To probe the chemical bonding state of SnO_2_, X-ray photoelectron spectroscopy (XPS) measurement was conducted for the c-SnO_2_, SnO_2_ NPs, and SnO_2_ QDs films on Silicon substrates. Figure S11 shows the O 1*s* core level signals from 2AT-SnO_2_ QDs and commercial SnO_2_ NPs, the O 1*s* peaks can be deconvolved into two individual peaks. The peaks at lower and higher binding energy were originated from Sn–O–Sn backbones serving as electron conductance pathways and the hydroxide species (Sn–OH) acting as shallow trap sites^34,55^. Accordingly, the 2AT SnO_2_ QDs exhibit a lower impurity level compared to the benchmark commercial SnO_2_ NPs. It is suggested that hydroxide species (shallow trap sites) can be effectively suppressed with the presence of 2AT within the SnO_2_. As demonstrated in Fig. 3b, we observe strong peaks at 487.5 and 495.8 eV, which are attributed to Sn^4+^ ion, and thus suggest the formation of SnO_2_. Meanwhile, the photoelectron binding energy of Sn 3*d* core level for SnO_2_ QDs film exhibit a blue chemical shift (toward high binding energy) in contrast to the c-SnO_2_ and SnO_2_ NPs film, indicating that an effective chemical bonding due to the presence of 2AT additive. In addition, we identified the core level signal of N1*s*, S2*p*, and Br3*d*, originating from the multi-functional groups of 2AT additive. Notably, N1*s* peak at 400.5 eV (Fig. S12A) from SnO_2_ QDs film is associated with the pronated –NH^3+^ terminal group of 2AT additive^48,56^, featuring successful chemical bonding of amino (or 2-aminoethyl) groups via Sn–N bond onto the surface of SnO_2_ QDs. Similarly, the signal of the S 2*p* core level (Fig. S12B) appears obviously in comparison with the c-SnO_2_ and SnO_2_ NPs film, which can be located at roughly 165.2 eV. This is generally assigned to the C–S–C (sulfide) or C–S–H (thiol)^57,58^, indicating the presence of sulfur species functionalities on SnO_2_ QDs. Besides, we also detected the characteristic signals of Br 3*d* core level located at a binding energy of 68.3 eV from SnO_2_ QDs (Fig. S12C). It is presumable that the peak could be responsible for the Sn–Br–O and Br–O state^35,59^, revealing that the Br ions from the 2AT additive are bonded to the Sn atoms of SnO_2_ QDs. We therefore indicate that SnO_2_ QDs were indeed equipped with multi-functional terminal groups, possibly by chemical interaction or surface anchoring. Based on the success of ligands-anchored SnO_2_ QDs, we proposed that multi-functional terminal groups of ligands-anchored SnO_2_ QDs effectively passivate interfacial imperfections^36,38^, such as deep level traps (e.g., uncoordinated Pb^2+^, Pb-I antisite defects, uncoordinated halide ion) and shallow level traps (e.g., halide vacancies and organic species vacancies), via coordinate bonding and ionic (hydrogen) bonding. As shown in Fig. 3a, organic amine terminal groups (a primary amine and a formamidine) of 2AT not only can occupy A-site vacancies (shallow level traps) but also passivating the undercoordinated Pb^2+^ or Pb-I antisite defects (deep level traps) at the interface of ETL/PVSK. Furthermore, the terminal –NH^3+^ cation of 2AT can passivate negatively charged defects or dangling bonds (uncoordinated halide ion) at the interface through ionic (hydrogen) bonding. In addition, sulfur species of ligand-tailored SnO_2_ QDs could afford interface passivation via coordinate bonding between uncoordinated Pb^2+^ and sulfur atom^57^. Exploiting the significantly beneficial effects of ligands-anchored SnO_2_ QDs on interface binding, high-quality perovskite film with efficient device performance can be achieved, as we will discuss below.

First, we examine the chemical bonding between 2AT-SnO_2_ QDs and perovskite –Pb 4*f* spectra of the 2AT-SnO_2_ QDs/perovskite and the SnO_2_ NPs/perovskite samples were investigated as demonstrated in Fig. S13. There are two main peaks Pb 4*f*_7/2_ and Pb 4*f*_5/2_ located at 138.76 and 143.61 eV, respectively. We attributed the two small noticeable peaks at 136.85 and 141.74 eV to the presence of metallic Pb, likely due to the existence of non-radiative recombination centers of commercial SnO_2_ NPs-based perovskite which is detrimental to device performance^60^. It is noted that the Pb 4*f*_7/2_ and Pb 4*f*_5/2_ of the 2AT-SnO_2_ QDs/perovskite sample show lower binding energies of 138.57 and 143.47 eV, respectively. This is probably due to the fact that Pb ion accepts electron pairs from the functional groups within 2AT via coordination bonding^61^. These chemical interactions significantly passivate the trap states, resulting in a long carrier lifetime and suppressed hysteresis. Moreover, we further increase the concentration of 2AT by five times in the SnO_2_ QDs, the Pb 4*f* peaks are further shifted by 0.35 eV towards the lower binding energy direction. This suggests that the ligands of 2AT in the SnO_2_ QDs have a stronger coordination interaction with the subsequent perovskite. In addition, partial ligands on SnO_2_ QDs surface could exchange or wash away during the coating of perovskite precursor, and these detached ligands contribute to the passivation in the bulk perovskite^31^.

Ultraviolet photoelectron spectroscopy (UPS) and UV–visible absorption spectra measurement were thoroughly conducted in order to determine the energy band diagram of ETLs and PVSK films, and the detailed results are summarized in the Supporting Information (Figs. S14, S15 and Table S1). We found that the energy-level diagram of the device with our ligand-tailored SnO_2_ QDs ETL exhibits a more favorable band alignment for charge transfer among these three ETLs. Capacitance–voltage measurements were conducted to further elucidate the effect of various ETLs on strengthening the built-in potential (*V*_bi_) in PSCs by Mott–Schottky analysis (Fig. S16). Notably, an enhanced built-in potential enables a reinforced built-in field that efficiently sweeps out the photo-generated carriers. Importantly, this indicates that the separation of photo-generated carriers in SnO_2_ QDs based PSCs is more efficient and the reinforced *V*_bi_ provide more efficient driving force for the improvement in PCE.

To investigate the impact of multi-functional groups in SnO_2_ on the perovskite crystallization process, we first check the contact angles (Fig. 3c) of various ETLs-coated FTO substrates. The contact angle is a direct signature of the surface wettability which largely affects the surface nucleation density and Gibbs free energy for nucleation. That is, a lower contact angle value gives rise to a reduced Gibbs free energy, thus enabling a better nucleation process^25^. Taking c-SnO_2_ (43.7°) and SnO_2_ NPs (14.5°) coated FTO substrates as a comparison, we find that the SnO_2_ QDs sample shows the lowest contact angle (5.8°), which can be ascribed to the nature of hydrophilic organic amine terminal groups at the ligand-tailored SnO_2_ QDs. These results indicate that the SnO_2_ QDs sample effectively promotes a higher perovskite nucleation density which is responsible for an improved crystallization process resulting in a better crystallinity and enhanced interface physical binding (Figs. S17 and S18A). Therefore, we hypothesize that functional organic amine terminal groups of the ligands-armed SnO_2_ QDs possess the ability to act as a seeding-controlling agents to alter the crystallization kinetics resulting in improved crystallinity and favorable PVSK surface feature (Fig. S17D–L). To verify our hypothesis, we further carried out grazing incident wide-angle X-ray scattering (GIWAXS) to examine the quality of perovskite films. Figure 3d shows 2D GIWAXS patterns of perovskite films on the ETLs-coated quartz substrates, all the sample displays the typical scattering rings with a *q*_r_ value (Fig. 3e) of 1.0, 2.0, and 2.2 Å^−1^ which corresponding to the (110), (220), and (310) planes^62^. It is noted that SnO_2_ QDs sample clearly demonstrates the highest diffraction (110) peak intensity at *q*_r_ = 1.0 Å^−1^ compared to the other two samples, suggesting that a better crystallinity. To quantitatively estimate the non-radiative recombination in our devices, we tested our solar cells operating as LEDs under different voltage bias as shown in Fig. 3f and a bright red emission (~770 nm) can be observed (inset). Figure S18B suggests that the SnO_2_ QD-based PSCs as LED exhibit the lowest turn-on voltage over other two samples due to the reduced interfacial energy losses from carrier injection and transport^63^. Figure 3g shows the plot of ELQE as a function of current density. With a higher maximum ELQE (ELQE_max_) of 4.4%, the SnO_2_ QD-based PSCs deliver a lower nonradiative *V*_oc_ loss (∆*V*_oc, nonrad_) of 133 mV using ELQE @*J*_SC_ injection (0.61%) according to literature^64^. In comparison, the ∆*V*_oc, nonrad_ (ELQE_max_ & ELQE_*J*sc injection_) of c-SnO_2_ and SnO_2_ NPs based PSCs are ~187 mV (0.6% and 0.08%) and ~162 mV (1.3% and 0.12%), respectively. Detailed results are listed in the Supporting Information (Table S2). These results indicate that SnO_2_ QD-based samples effectively minimize the non-radiative recombination centers at the ETL/perovskite interface compared to other samples, resulting in an increased EL efficiency. Owing to the reduced interfacial energy loss, noticeably *V*_oc_ improvement (~0.05 V) can be attained for SnO_2_ QD-based samples.

**Fig. 4:**
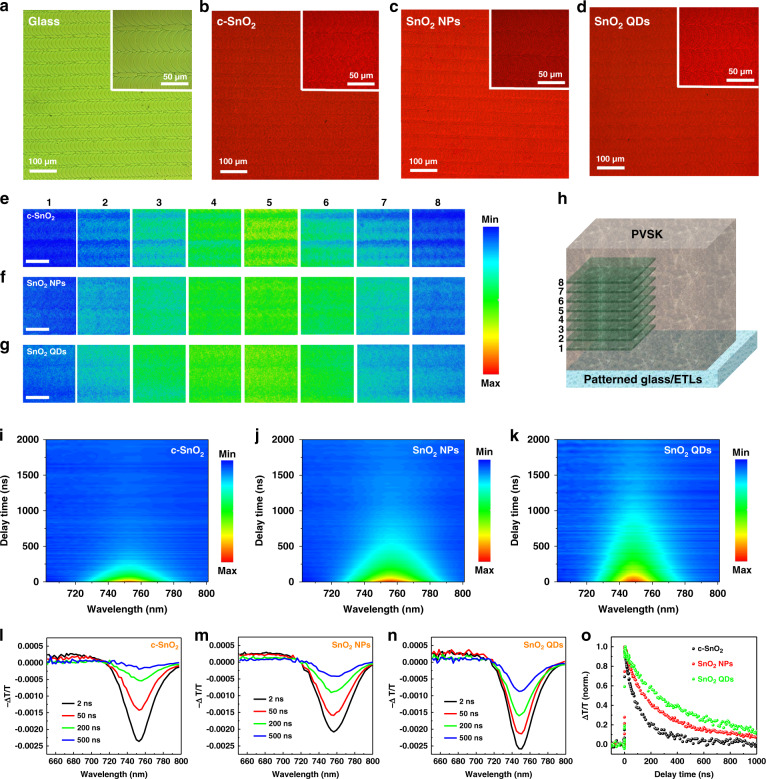
Local investigations on PVSK thin films on various substrates. Optical microscopy images of **a** patterned glass, **b** patterned glass/c-SnO_2_/PVSK, **c** patterned glass/SnO_2_ NPs/PVSK, and **d** patterned glass/SnO_2_ QDs/PVSK. Insets are the corresponding high magnification images. Spatial confocal luminescence microscope screenshot at *λ*_excitation_ = 633 nm of **e** patterned glass/c-SnO_2_/PVSK, **f** patterned glass/SnO_2_ NPs/PVSK, and **g** patterned glass/SnO_2_ QDs/PVSK. Scale bar is 50 µm. **h** Schematic profile of depth-dependent spatial confocal luminescence image acquired from different regions (level 1–8) in the *Z* direction. **i**–**k** 2D contour plots of transient absorption (TA) results pumped by 532 nm with fluence as low as 0.27 µJ cm^−2^. **l**–**n** representative TA spectra at selected delay times. **o** Normalized decay dynamics of ground-state-bleach signals of each sample. TA measurements are performed under room temperature and samples are protected by nitrogen gas

To further investigate the effect of different ETLs on the film quality of PVSK film under the micro-scale, we used confocal fluorescence microscopy to map the spatially resolved luminescence generation in these PVSK thin films, which allows us to visually analyze the microscale spatial heterogeneity of as-prepared PVSK films. As a correlative scan area, we start with an optical microscope image of the samples, this allows direct comparison between optical microscope images and confocal photoluminescence (PL) images. In Fig. 4a, we show the optical microscopy image of laser-patterned glass substrates, which suggest a regular squama-like textured feature (~50 μm width) row in a row. Figure 4b–D shows the optical microscopy of patterned glass/c-SnO_2_/PVSK, patterned glass/SnO_2_ NPs/PVSK, and patterned glass/SnO_2_ QDs/PVSK. Under an optical microscope, similar squama-like textured features were observed in the PVSK films upon the various ETLs coated patterned glass substrates. Figure 4e, f show the 120 µm-by-120 µm spatial confocal PL images of patterned glass/c-SnO_2_/PVSK, patterned glass/SnO_2_ NPs/PVSK, and patterned glass/SnO_2_ QDs/PVSK, which were acquired from different depth regions (Fig. 4h, level 1–8) within the perovskite layer with respect to the ETLs. We found that PL intensity becomes notably weaker when the detected region approach to the PVSK/ETLs interface and/or PVSK top surface, which is attributed to the quenching effect originated from carrier injection of SnO_2_ ETLs, recombination centers of PVSK/ETLs interface and PVSK surface defects (or strain-related defects), for example, dangling bonds, undercoordinated ions and dislocations^36,38^. We also observed that PL intensity of the c-SnO_2_ sample gives the weakest signal in the shallow probing depth (detected region: level 8), indicating that more surface defects are induced in the c-SnO_2_/PVSK sample, resulting in a significant PL quenching compared to SnO_2_ NPs/PVSK and SnO_2_ QDs/PVSK samples. This result also indicates that incorporation of ligands-functionalized SnO_2_ QDs ETL plays a role both in more efficient interface charge transfer and strengthening the overall passivation of non-radiative recombination, delivering an improved PVSK film quality. It is interesting to point out that in the confocal PL mapping image of patterned glass/c-SnO_2_/PVSK sample, we observed dim stripes pattern as well as a lot of heterogeneities, which are the evidence of non-radiative recombination centers^65^. In contrast, the PL intensity from SnO_2_ NPs/PVSK and SnO_2_ QDs/PVSK sample demonstrates a much more uniform and intense PL signal distribution. We attribute this dim stripe patterns along with the significant PL quenching to the increased physical defects (or non-radiative recombination center), which are located at the grooves between the two squama-like textured feature rows. This is due to the fact that c-SnO_2_/PVSK sample exhibits poor groove (or valley) filling ability. On the basis of the above results, it is well-confirmed that ligand-tailored SnO_2_ QDs are able to deliver a physical defects-free interface, suppressed non-radiative recombination, reduced PVSK surface defects, and provide a better seeding template to assist in the nucleation and subsequent perovskite growth.

To further explore the dynamics of charge carriers, perovskite films on various ETLs prepared on quartz substrates were investigated by nanosecond broadband transient absorption (TA) spectroscopy. 2D contour plots of the TA data are shown in Fig. 4i–k, and representative TA spectra at different delay times are presented in Fig. 4l–n. All TA spectra feature a negative signal of Gaussian shape around 750 nm. This band is consistent with published data and has been attributed to ground state bleaching (GSB) as a consequence of the population of band edge states by charge carriers^66,67^. We note that in the case of SnO_2_ QDs, the GSB signal exhibits a smaller full width at half maximum (FWHM). Since the GSB originates from charge filling of band edge states, its width is related to the charge distribution profile^67^. In fact, a narrower FWHM indicates less imperfections, such as defects or traps, which in turn leads to a higher *V*_oc_^68^. In addition, SnO_2_ QDs/perovskite also exhibit a blue-shifted GSB (~3 nm) compared to the other two samples. This is attributed to an optimized degree of order (the same underlying mechanism leading to a narrower FWHM of GSB) and accordingly the suppression of non-radiative centers, as revealed from the decay kinetics in Fig. 4o. Since the GSB originates from the population of band-edge states by charge carriers, investigating decay dynamics provides insight to the underlying charge recombination mechanisms. Here, we find that all decays can be described by single exponential functions with lifetimes of 112.3 ± 2.5, 227.3 ± 2.5, and 405.1 ± 4.6 ns, respectively. In view of the proposed mechanism^69^, we thus assign the recombination to be of first-order. Considering the weak excitonic binding energy in these perovskite materials, we can further assign the first-order recombination to trap-assisted recombination, that is, Shockley–Read–Hall (SRH) recombination. Hence, the increased lifetime indicates that the trap density is the highest in the c-SnO_2_ sample, and decreases in the commercial SnO_2_ NPs sample, and is the lowest in the ligand-assisted SnO_2_ QDs sample. The observation of a decreasing trap density implies that multifunctional terminal groups in the SnO_2_ can effectively passivate the interface and improve the film quality, both of which contribute to the solar cell performance. These findings consistently explain the alleviated (or negligible) hysteresis and enhanced *V*_OC_ in the SnO_2_ QDs-based device, which are further supported by 2D GIWAXS patterns, ELQE, and spatial confocal PL microscopy as discussed above.

### Solar cell performance and device stability

**Fig. 5:**
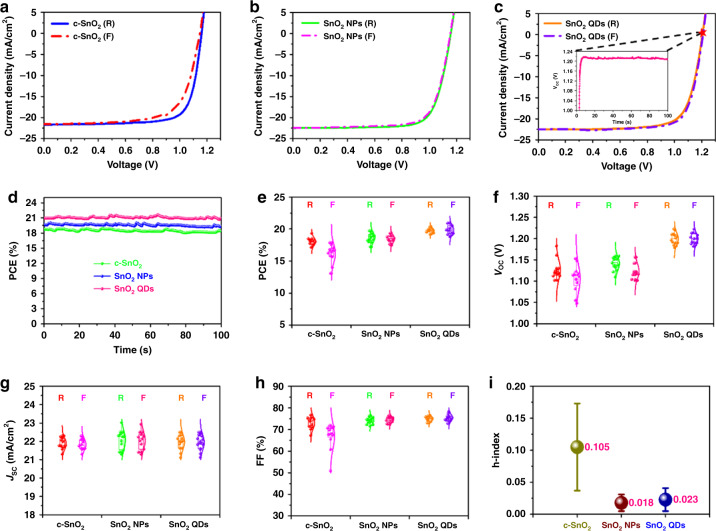
Photovoltaic performance of PSCs. Best-performing *J*–V curves with reverse and forward scans of PSCs with **a** c-SnO_2_ ETLs, **b** SnO_2_ NPs ETLs, and **c** SnO_2_ QDs ETLs. Inset: stabilized *V*_oc_ as a function of the time. **d** Stabilized PCEs at the maximum power point under simulated solar illumination (AM1.5 G, 100 mW cm^−2^). Histograms of the device photovoltaic parameters **e** *V*_oc_, **f** *J*_sc_, **g** FF, and **h** PCE obtained from a batch of PSCs with different ETLs. **i** Average h-index with standard deviation extracted from *J*–*V* curves of PSCs with various ETLs

Previous studies reveal that perovskite shows improved structural stability, suppressed ion migration, reduced defect density, and lowered voltage-loss by doping a small amount of alkali cations^70^, such as cesium (Cs) rubidium (Rb), and potassium (K). In this study, as an example, we adopted the state-of-the-art Cs, Rb, and K co-doped FAMA mixed-cation perovskite with a one-step chlorobenzene anti-solvent quenching technique^71^. The *J*–*V* curves of the best-performing c-SnO_2_, SnO_2_ NPs, and SnO_2_ QDs based PSCs under the RS and forward scan (FS) directions are shown in Fig. 5a–c, with the corresponding photovoltaic parameters summarized in Table S3. The c-SnO_2_-based devices exhibit a PCE of 17.6% (FS) and 19.3% (RS) with considerable *J*–*V* hysteresis, whereas PCEs for devices with SnO_2_ NPs (FS: 19.3%, RS: 19.8%) and SnO_2_ QDs (FS: 21.0%, RS: 20.4%) have negligible hysteresis. The SnO_2_ QDs-based devices exhibit a remarkable improvement in *V*_oc_ (FS: 1.208 V, RS: 1.205 V) compared to the c-SnO_2_-based devices (FS: 1.148 V, RS: 1.161 V) and SnO_2_ NPs-based devices (FS: 1.159 V, RS: 1.156 V). The SnO_2_ QDs-based devices also have stable *V*_oc_ under 1 sun illumination (inset in Fig. 5c). This dramatic enhancement in *V*_oc_ is likely ascribed to reduced interfacial non-radiative recombination and improved PVSK film quality. This indicates that the ligand-tailored SnO_2_ QDs, which possess multi-functional terminal groups, play an essential role in effective interfacial passivation, hammered by the observations in 2D GIWAXS, ELQE, spatial confocal PL microscopy, and nanosecond-TA spectroscopy. Figure 5d further confirms the stable power output during maximum power point (MPP) tracking under 1 sun illumination (100 s) for c-SnO_2_ (18.8%), SnO_2_ NPs (19.7%), and SnO_2_ QDs (21.2%) based devices, respectively. To examine the effect of these ETLs on the device-to-device reproducibility variation, we fabricated 38 independent devices incorporating three different ETLs and statistically summarized the PV parameters. Figure 5e clearly depicts that the performances of PSCs with commercial SnO_2_ NPs ETLs (FS: 18.47 ± 0.61%, RS: 18.67 ± 0.72%) surpass those with c-SnO_2_ ETLs (FS: 16.17 ± 1.40%, RS: 18.05 ± 0.58%), while the PSCs with SnO_2_ QDs ETLs (FS: 19.77 ± 0.38 %, RS: 19.87 ± 0.71%) have the best performances. The improved PCE is mainly attributed to an increase in the *V*_oc_ of ~50 mV with SnO_2_ QDs ETLs comparing to the PSCs with the other two ETLs. The c-SnO_2_ ETL-based PSCs have a much larger standard deviation, as shown in Fig. 5f. Short-circuit current density (Fig. 5g) obtained from c-SnO_2_-based devices (FS: 21.83 ± 0.03 mA cm^−2^, RS: 21.87 ± 0.03 mA cm^−2^) is smaller than those obtained from SnO_2_ NPs-based devices (FS: 22.05 ± 0.50 mA cm^−2^, RS: 22.06 ± 0.53 mA cm^−2^) and SnO_2_ QDs-based devices (FS: 21.94 ± 0.41 mA cm^−2^, RS: 21.97 ± 0.41 mA cm^−2^). The low performance of c-SnO_2-_based samples is linked to poor interfacial physical contact. This configuration exhibits the most exaggerated interfacial physical defects (Figs. 2a, S10B, and S17A), which can act as carrier recombination centers resulting in lower *J*_sc_ as well as decreased FF (Fig. 5h). Expectedly, the average h-index (Fig. 5i) obtained from *J*–*V* characteristics in c-SnO_2_-based devices (0.105 ± 0.068) is much larger than those of PSCs with SnO_2_ NPs (0.018 ± 0.013) and SnO_2_ QDs (0.023 ± 0.018). Previous research works have established that *J*–*V* hysteresis mainly originates from the existence of traps/defects on the surface of ETLs and PVSK bulk films as well as their interfaces^36–38^. According to the above results, we can deduce that the dramatic suppressed *J*–*V* hysteresis should result from decreased interfacial physical defects and reduced charge accumulation at the interface, as well as improved PVSK film quality.

It worth mentioning that our multi-functional terminal groups anchored SnO_2_ QDs ETLs have strongly reduced annealing temperature—even to below 100 °C. The devices with room temperature processed ETL still achieve an acceptable performance (FS: 15.1 ± 1.9%, RS: 15.6 ± 1.7%). We designate the differentiated annealing temperature of SnO_2_ QDs ETLs from 200 °C to room temperature (best-performing PSCs are shown in Fig. S19), and the corresponding statistical data of the photovoltaic parameters are shown in Fig. S20. It is very encouraging that the 100 °C-annealed ETL device still shows high performance (FS: 18.7 ± 0.99%, RS: 18.5 ± 0.79%), only slightly lower than that of 120 °C-annealed ETL samples (FS: 19.0 ± 0.68%, RS: 19.1 ± 0.78%). The optimal condition is achieved with samples based on 150 °C-annealed ETLs (FS: 19.9 ± 0.71%, RS: 19.8 ± 0.38%) while devices based on 200 °C-annealed ETLs exhibit a slightly smaller PCE value (FS: 19.9 ± 0.88%, RS: 19.3 ± 0.62%).

We then systematically investigate the stability of our as-prepared unencapsulated devices (c-SnO_2_, SnO_2_ NPs and SnO_2_ QDs), which were carried out under a series of aging conditions (e.g., dry box storage with 25–30% relative humidity, 85 °C thermal aging in glovebox, and continuous UV light irradiance aging in the glovebox). The results of stability tests were shown in Fig. S21. The storage lifetime (dry box with 25–35% relative humidity) of PSCs with SnO_2_ NP ETLs or SnO_2_ QD ETLs exhibited a more significant enhancement compared to that of c-SnO_2_-based devices. The c-SnO_2_-based devices maintain only 48% of their initial value after 1000 h degradation, while PSCs with SnO_2_ NP ETLs or SnO_2_ QD ETLs retaining ~76% and ~80% of their initial PCEs, respectively. The PSCs with SnO_2_ QD ETLs (or SnO_2_ NP ETLs) showed improved thermal (Fig. S21B) and UV irradiance stability (Fig. S21C), which allowed the devices to achieve a *T*_80_ (when PCE drops to 80% of the original value) of ~180 h after 85 °C thermal aging testing or ~40 h after 365 nm UV irradiance aging testing, while the c-SnO_2_-based devices only demonstrated a *T*_80_ of ~50 h with 85 °C thermal aging and ~20 h with 365 nm UV irradiance aging. These results possibly indicate that improved physical contact at FTO/ETLs interface (or ETLs/PVSK interface), and thereby reduced interface nonradiative recombination accounts plausibly for the observed enhanced thermal, UV, and moisture endurance. Furthermore, strengthened interfacial adhesion and efficient interfacial passivation could be another main reason for the improved stability of devices with tailored SnO_2_ QDs.

**Fig. 6:**
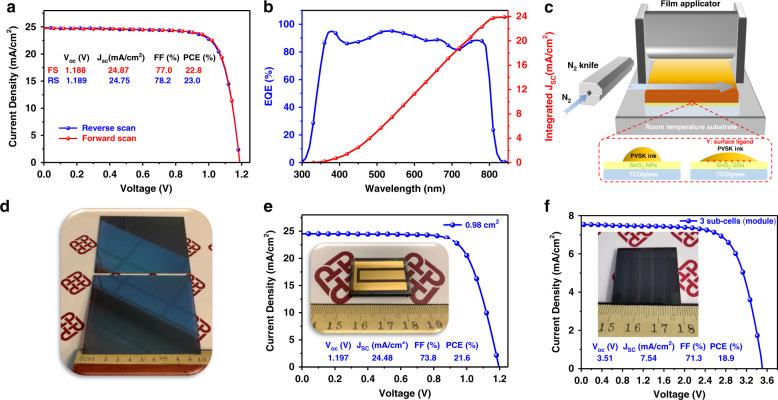
Best cell and mini-module performance of blade-coated devices. **a** *J*–*V* curves, **b** EQE spectra, and integrated current density of the best SnO_2_ QDs-based perovskite solar cell with a bandgap of 1.541 eV. **c** Schematic illustration of air-knife-assisted blade-coating at room temperature. **d** Digital image of the blade-coated large area (90 mm × 100 mm) perovskite film on SnO_2_ QDs-coated substates. **e** *J*–*V* curves of the best performance blade-coated devices with SnO_2_ QDs. Inset: a digital image representing a 20 × 30 mm^2^ substrate (active area: 0.98 cm^2^) by meniscus blade-coating. **f** *J*–*V* curves of a champion module with a 1.613 eV perovskite system. Inset: a digital image representing a 30 × 30 mm^2^ substrate with three sub-cells (a total active area of 2.7 cm^2^)

To further prove the versatility and genericity of the ultrafine SnO_2_ QDs ETL, we applied this new ETL to a lower bandgap, i.e., 1.541 eV (CsFAMA) perovskite system, via manufacturing friendly blade coating process and the results are even more exciting. A high PCE (reverse scan) of 23.02% (certified at 22.51%, Enli Tech., Fig. S22) was realized for the blade-coated n–i–p devices (Fig. 6a, b and Table S4). Figure 6c shows our setup of room temperature meniscus-guided blade-coating technique assisted with nitrogen air-knife quenching^72^. The perovskite precursor solution was blade-coated on the ligand-tailored SnO_2_ QDs coated substrate by an automatic wire-bar coater with an adjustable film applicator, delivering a large-area (up to 90 mm × 100 mm here) high-quality perovskite film (Fig. 6d). In this study, upscaling device with an active area from 0.04 to 0.98 cm^2^ exhibits a record performance (21.6% PCE, maximum *V*_OC_ of 1.205 V) for SnO_2_ ETL-based upscaling PSCs (Fig. 6e). Considering the perovskite bandgap of 1.541 eV, this indicates a significantly small bandgap–*V*_OC_ offset (*W*_OC_ = *E*_g_–*V*_OC_ or simply *V*_OC_ loss) of 0.336 V, representing one of the lowest *V*_OC_ loss for SnO_2_ ETL-based PSCs. We further extended it to blade coated 30 × 30 mm^2^ mini-modules with 1.613 eV perovskite systems (18.9% PCE for three sub-cells, Figs. 6f and S23) and 1.541 eV (19.5% PCE for two sub-cells, Fig. S24A). More noticeably, after delicate optimization, a substantially enhanced PCE (*V*_OC_) from 20.4% (1.152 V) to 22.8% (1.242 V, 90 mV higher *V*_OC_) was achieved in the meniscus-guided blade-coated PSCs (1.613 eV perovskite system, Figs. S24B and S24C), via replacing the benchmark commercial colloidal SnO_2_ ETL1 with our ligand-tailored SnO_2_ QDs ETL2 (Fig. S24D). Detailed device PV parameters are summarized in Table S4, Figs. S25, and S26.

## Discussion

In this paper, we demonstrate a facile, ligand-assisted, and room-temperature synthetic approach to rapidly formulate SnO_2_ QDs anchored by multi-functional terminal groups and utilize such SnO_2_ QDs as ETLs to in situ manipulate critical interfacial contact in planar perovskite solar cells. We explore the interfacial contact and visualize morphological properties via systemic characterization to in-depth illuminate the synergistic effect of physical and chemical interfacial modulations, originated from the new multifunctional ligand-tailored SnO_2_ QDs, on enhanced device performance. Compared with the existing benchmark SnO_2_ nano-materials, ligand-tailored SnO_2_ QDs exhibited superior properties, including low processing temperature (<100 °C), strengthened perovskite interfacial physical and chemical adhesions, buried interface defects passivation, and preferred perovskite crystallization-directing. These benefits together allow us to achieve a PCE of 23.02% (certified efficiency 22.51%) in a 1.541 perovskite system (RS, 0.04 cm^2^ active area), with significantly enhanced EL quantum efficiency thanks to the substantially suppressed non-radiative recombination and buried interface defects. Furthermore, we successfully prove the scalability of new ETL in the blade-coated PSCs—0.98 cm^2^ active area PSC with 21.6% PCE (a *V*_OC_ loss as low as 0.336 V)—a record for SnO_2_ ETL-based upscaling PSCs. Moreover, in the blade-coated 1.613 eV PSCs system, a substantially enhanced PCE (*V*_OC_) from 20.4% (1.152 V) to 22.8% (1.242 V, 90 mV higher *V*_OC_) has been achieved via replacing the benchmark commercial colloidal SnO_2_ ETL with our new ETLs. Moreover, encouraging mini-modules were successfully demonstrated in both 1.541 eV (19.5% PCE for two sub-cells) and 1.613 eV (18.9% PCE for three sub-cells) perovskite systems. Our in situ solution chemistry engineering of metal oxide synthesis contrives a new direction toward achieving low temperature and high quality functionalized SnO_2_ ETLs, compatible with upscaling manufacture of large-area high-quality perovskite films for solar cells and other optoelectronic devices.

## Materials and methods

Experimental details can be found in the Supplementary Information.

## Supplementary information

supporting information

## Data Availability

All data is available in the main text or Supplementary materials.
