# Supplementary material for: Room-temperature multiple ligands-tailored SnO2 quantum dots endow in situ dual-interface binding for upscaling efficient perovskite photovoltaics with high VOC
Source: Light Sci Appl. 2021 Dec 2;10:239. doi: 10.1038/s41377-021-00676-6 (PMC8639768; doi:10.1038/s41377-021-00676-6)
Supplement: Supplementary file 1 — supporting information [file 41377_2021_676_MOESM1_ESM.docx]

Supplementary Information for

Room-Temperature Multiple Ligands-Tailored SnO_2_ Quantum Dots Endow *in situ* Dual-Interface Binding for Upscaling Efficient Perovskite Photovoltaics with High *V*_OC_

Zhiwei Ren^1,6,10^, Kuan Liu^1,9,10^, Hanlin Hu^8,^*, Xuyun Guo^2,7^, Yajun Gao^3^, Patrick W.K. Fong^1^, Qiong Liang^1,9^, Hua Tang^1^, Jiaming Huang^1^, Hengkai Zhang^1^, Minchao Qin^4^, Li Cui^1^, Hrisheekesh Thachoth Chandran^1^, Dong Shen^5^, Ming-Fai Lo^5^, Annie Ng^6^, Charles Surya^6^, Minhua Shao^7^, Chun-Sing Lee^5^, Xinhui Lu^4^, Frédéric Laquai^3^, Ye Zhu^2^, Gang Li^1,9,^*

^1^Department of Electronic and Information Engineering, Research Institute for Smart Energy (RISE), Guangdong-Hong Kong-Macao (GHM) Joint Laboratory for Photonic-Thermal-Electrical Energy Materials and Devices, The Hong Kong Polytechnic University, Hung Hom, Kowloon, Hong Kong, China.

^2^Department of Applied Physics, The Hong Kong Polytechnic University, Hung Hom, Kowloon, Hong Kong, China.

^3^King Abdullah University of Science and Technology (KAUST), KAUST Solar Center (KSC), Physical Sciences and Engineering Division (PSE), Material Science and Engineering Program (MSE), Thuwal, 23955-6900, Kingdom of Saudi Arabia.

^4^Department of Physics, The Chinese University of Hong Kong, Shatin 999077, Hong Kong, China.

^5^Center of Super-Diamond and Advanced Films (COSDAF), Department of Chemistry, City University of Hong Kong, Hong Kong, China.

^6^Department of Electrical and Computer Engineering, Nazarbayev University, Nur-Sultan, Kazakhstan.

^7^Department of Chemical and Biological Engineering, The Hong Kong University of Science and Technology, Clear Water Bay, Kowloon, Hong Kong, China.

^8^Hoffmann Institute of Advanced Materials, Shenzhen Polytechnic, 7098 Liuxian Boulevard, Shenzhen 518055, China.

^9^The Hong Kong Polytechnic University Shenzhen Research Institute, Shenzhen 518057, China

^10^These authors contributed equally to this work.

*Correspondence: [hanlinhu@szpt.edu.cn](mailto:hanlinhu@szpt.edu.cn) (H.H.); [gang.w.li@polyu.edu.hk](mailto:gang.w.li@polyu.edu.hk) (G.L.)

**Materials.**

Chlorobenzene, dimethylformamide, dimethyl sulfoxide, 4-*tert*-butylpyridine, acetonitrile, Li-TFSI lead (II) bromide (99.99%), cesium iodide, rubidium iodide, and potassium iodide were purchased from Sigma-Aldrich; Formamidine iodide, methylammonium bromide were purchased from Greatcell Solar. 2-(2-aminoethyl) isothiourea dihydrobromide, tin (IV) oxide (15% in H_2_O colloidal dispersion) and tin (II) chloride dihydrate were purchased from Alfa Aesar; Lead (II) iodide (99.998%) was purchased from TCI and spiro-OMeTAD was purchased from Lumtec;

**Preparation of c-SnO_2_, SnO_2_ NPs and SnO_2_ QDs precursor.**

A 0.1 M c-SnO_2_ precursor was prepared by dissolution of SnCl_2_ 2H_2_O in anhydrous ethanol, followed by a 60 min vigorous stirring. SnO_2_ NPs precursor was prepared by adding tin (IV) oxide (15% in H_2_O colloidal dispersion) into DI water with a volume ratio of 1:5. Different concertation (Figure S27) of SnO_2_ QDs precursor (0.1 M, 0.13 M, 0.16 M, and 0.2 M) were prepared by dissolving 2-(2-aminoethyl) isothiourea dihydrobromide and SnCl_2_ 2H_2_O in DI water with a fixed molar ratio of 1: 6.5, followed by several hours (2 h at least) vigorous stirring under an oxygen rich ambient at room temperature. We find that 0.13 M SnO_2_ QDs-based PSCs exhibit optimal device performance.

**Preparation of perovskite precursor and perovskite films.**

CsI stock solution (1.5 M) in DMSO, RbI stock solution (1.5 M) in DMF: DMSO (4:1) and KI stock solution (1.5 M) in DMF: DMSO (4:1) were prepared. 1.61 eV-perovskite film was prepared through an anti-solvent one-step coating or room-temperature air-knife assisted blade-coating method, a stock solution of FAI: MABr: MACl: PbBr_2_: PbI_2_ (molar ratio: 1.1:0.2: 0.5: 0.2: 1.2) in DMF: DMSO (4:1) and an appropriate volume stock solution of CsI (2.8 %): RbI (2.8 %): KI (1.8 %) was spin-coated at 1000 rpm for 10 s and 4000 rpm for 30s, 200 μL chlorobenzene was drop-casted in the last 10-15s. For blade-coated devices, an automatic wire-bar coater (RK PrintCoat Instruments, K paint type) was empolyed in a humidity-control ambient environment. Small droplet of perovskite ink was dripped on the substrate and swiped linearly by an adjustable film applicator (BEVS 1806B/100) at the speed of 10 mm s^-1^. The gap between the film applicator and substrate was set as 100 μm. A laminar nitrogen knife (Wells Corp., 150 mm width) was installed right next to the blade substrate with the flow at an angle of 20^o^ to the substrate. The as-prepared wet perovskite film was gas-quenched by the nitrogen knife with the fixed nitrogen blow rate of 40m s^-1^ (calibrated using the Testo 416 flowmeter), in order to remove the extra precursor solution and induce the precipitate of perovskite intermediate adducts. As-prepared perovskite films were later transferred onto the hotplate at 120 ℃ for 60 min in controlled ambient air (20%-30% relative humidity). 1.54 eV-perovskite film was deposited by a stock solution of FAI: MABr: MACl: PbBr_2_: PbI_2_ (molar ratio: 1.41:0.07:0.4:0.07:1.47) in DMF: DMSO (4:1), blended with 4.4% CsI stock solution. The 1.54 eV blade-coated perovskite films was deposited using the same method in 1.61 eV perovskite system.

**Device fabrication**

The patterned FTO or ITO glass substrates were ultrasonically cleaned by detergent, DI water, acetone, and isopropanol for 15 min, respectively. The cleaned substrates were further dried by a pure nitrogen gun and treated with UV-ozone for 20 min. To form an ETL layer, the different SnO_2_ solution (as described above) was spin-coated on the substrates to achieve the thickness of 30-50 nm (Figure 2), followed by heat-treatment at 200 ℃ for 60 min (c-SnO_2_), 150 ℃ for 30 min (SnO_2_ NPs) and different temperature (Figure S19 and S20) for 60 min (SnO_2_ QDs). After cooling down to room temperature, ETL-coated substrates were treated with UV-ozone for 10-20 min and then transferred into a nitrogen-filled glovebox. Deposition of perovskite films with a bandgap of 1.61 eV and 1.54 eV was discussed above. Afterward, spiro-OMeTAD in anhydrous chlorobenzene (80 mg mL^-1^) with with the additives of Li-TFSI (17.5 μL from a stock solution of 520 mg mL^-1^ in acetonitrile) and 29 μL of tBP and was deposited on the perovskite at 3000 rpm for 30 s. The prepared samples with spiro-MeOTAD were stored and oxidized overnight in a humidity-controlled ambient (<10% relative humidity). Finally, oxidized samples were transferred into a vacuum chamber for the deposition of Au (80 nm) electrode through a metal shadow mask. The active area of device was 0.06 cm^2^. Spiro-OMeTAD HTL was then spin-coated onto the perovskite film. And a 80 nm thick Au was evaporated using a metal shadow mask to pattern the active area. Note that device active area was confined by the crossed area of Au electrode and transparent electrode, which was precisely measured using optical microscopy. To confirm our results, we have verified our result in a third-party independent academic lab in Hong Kong with high reputation/publication record in photovoltaic field and found that the measurement results are consistent.

**Characterization**

**JV measurement.**

The J-V characteristics of the devices were measured by using a Keithley 2400 source meter unit under the calibrated solar simulator (Enli Technology Co. Ltd) equipped with an AM 1.5 filter. Note that the J-V curves were obtained from the range of 1.2 V to -0.2 V (both reverse scan and forward scan) with a delay time of 10 ms (Scan step size: 20 mV). Anti-reflection MgF_2_ films were used during the measurement. No preconditioning protocol were used.

**EQE measurement.**

It was measured by a QE-R3011 system from Enli Technology Co. Ltd. The devices were measured under the monochromatic light split from 300 nm to 900 nm with 10 nm step size.

**AFM and optical microscopy.**

AFM images were performed on Smart SPM 1000 (AIST-NT) and optical images were collected by Leica DM4000 B Digital Microscope.

**SEM.**

It was performed on a Crossbeam 540 SEM (Carl Zeiss).

**SEM-FIB.**

The cross-sectional sample was prepared by a multi-Beam SEM-FIB system (JEOL Model JIB-4501), operated under 30 keV Ga^+^, equipped with platinum (Pt) deposition cartridge. To minimize the ion beam damage, the interested area was protected by hundreds of nanometers carbon layer coating first. After that, a several micrometer thick platinum layer was deposited with using the gallium ion beam and then milled to <100 nm thick lamella gradually. At last, the thin lamella was ex-situ lifted out to the Quantifoil holey carbon Cu grid by glass needle.

**TEM/STEM and EELS.**

Transmission electron microscopy and scanning TEM (STEM) were performed using JEOL JEM-2100F TEM/STEM (Tokyo, Japan) operated at 200 kV. Spectrum imaging of electron energy-loss spectroscopy (EELS) was carried out under 200 kV accelerating voltage with a 13 mrad convergence angle for the optimal probe condition. Energy dispersion of 0.7 eV per channel and 21 mrad collection angle were set up for EELS, HAADF images were acquired with an 89 mrad inner angle simultaneously.

**XPS and UPS.**

Measurements were performed on a VG ESCLAB 220i- XL surface analysis system equipped with and a He-discharge lamp and a monochromatic Al Kα X-ray source (1486.6 eV).

**EL EQE measurement**

The device operating as a LED, recorded by a LED photo-luminescence quantum yield measurement system (Enli Tech LQ-100) equipped with Keithley 2400 Source Measure Unit.

**GIWAXS measurement.**

It was carried out with a Xeuss 2.0 SAXS/WAXS laboratory beamline using a Cu X-ray source (8.05 keV, 1.54 Å) and a Pilatus3R 300K detector. The incidence angle is 0.2°.

**Confocal PL mapping.**

It was carried out with an inverted confocal laser scanning microscope LSM 780 (Carl Zeiss). The perovskite thin films were excited by a 633 nm He-Ne laser (5 mW). During the measurement, main beam splitter of 633 nm was used to separate the beam and emission signals was collected between 641 and 758 nm. Dry 20x objective corrected for both flat-field and chromatic aberration were used (Zeiss 20x NA 0.8, Plan-Apochromat). Pixel size was 120 nm with a µs-level pixel dwell time (integration time).

**TAS measurement.**

Nano-second (ns) to micro-second (μs) transient spectroscopy is carried out using a home-built pump–probe setup.

(1) Generation of super-continuum white light as probe pulses.

The laser seed from an oscillator (Coherent Vitesse) is first amplified by a titanium: sapphire amplifier (Coherent LEGEND DUO, 4.5 mJ, 3 kHz, 100 fs). The amplified pulse is then split into three beams, and each of them has pulse energies of 1.0, 1.5 and 2.0 mJ, respectively. The 2.0-mJ pulse is directed into an optical parametric amplifier (Light Conversion, TOPAS Prime) attached with a non-collinear difference frequency generator (Light Conversion, NDFG). In the present transient measurement, we select 1300 nm pulse outputted from TOPAS and focus it on a calcium fluoride (CaF_2_) crystal to generate super-continuum white light. To protect the crystal, the crystal is mounted on a continuously moving stage. Finally, we could generate the super-continuum pulse spectrally ranging from 450 nm to 1000 nm, and this range can cover the main spectroscopic features of perovskite films.

(2) Generation of pump pulses

Pump pulses comes from a sub-ns Q-switched Nd: YVO laser (InnoLas picolo AOT). This laser has a fundamental wavelength of 1064 nm, and through the harmonic generator crystal (LBO) it can output the other three wavelegnths of 532 nm, 355 nm and 266 nm. For the current measurements, 532 nm is selected. In addition, the time delay is achieved electronically. The LEGEND DUO amplifier outputs a synchronized transistor-transistor logic (TTL) with the same repetition frequency of 3000 Hz. This TTL series is then connected to a ½ frequency dividor and chopped electronically. Afterwards, TTL series, with the repetition frequence of 1500 Hz, serve to trigger a delay generator (Stanford Research System, DG 535) which again triggers the AOT pump laser. The microsecond-long delay is achieved accordingly. In our microsecond transient setup, the jitter between pump and probe pulses is roughly 100 ps. So, the IRF is mainly determined by the pulse width of AOT laser which is in the sub-ns range.

(3) Acquisition of super-continuum white light

The light is guided to a custom-made prism spectrometer (Entwicklungsbüro Stresing) where the light is dispersed by a prism onto a 512-pixel negative channel metal oxide semiconductor (NMOS) linear image sensor (HAMAMATSU S8381-512). The acquired signal is read out at 3000 Hz. The spectral calibration is performed with a series of band-pass filters and the spectral gap between adjacent filters is around 50 nm. To make the signal reading as accurate as possible, we also take into account both dark current as well as the scattered light from pump.

(4) Evaluation of probe/pump sizes

The size of probe/pump pulses are measured by a beam profiler. When fitting the intensity profile with Gaussian function, the pulse diameter corresponds to the case where 86% energy is transmitted. In the present measurements, the diameters of probe and pump pulses are tuned to be around 0.5 and 1.0 mm, respectively. This guarantees that the pump pulse is sufficiently larger than the probe pulse.


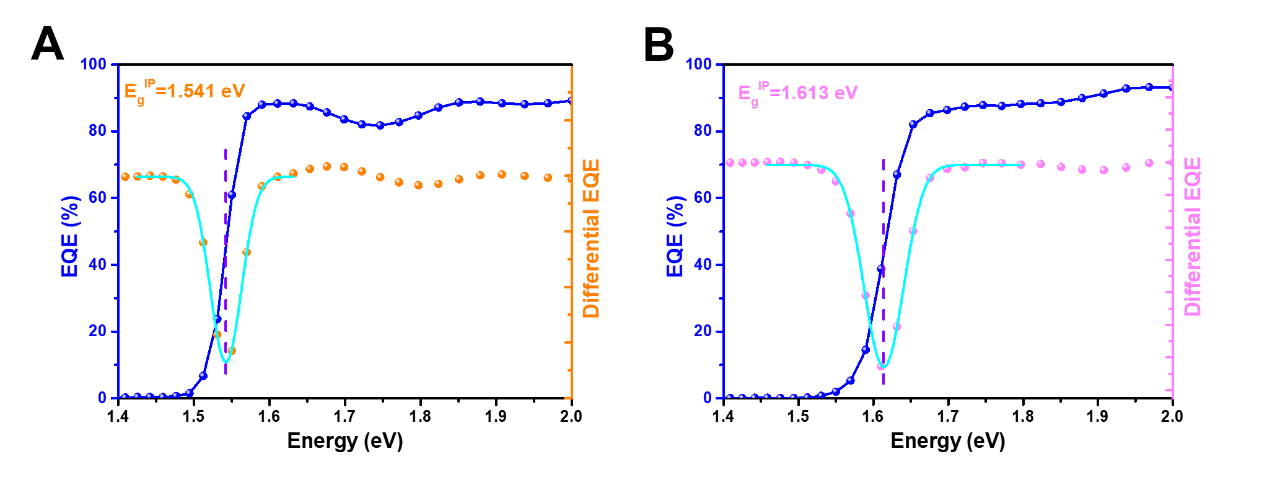


**Figure S1.** Calculation of the bandgap of 1.54-perovskite (a) and 1.61-perovskite (b) through EQE spectra. *E_g_^IP^* can be calculated by the maximum differential value of EQE.

**
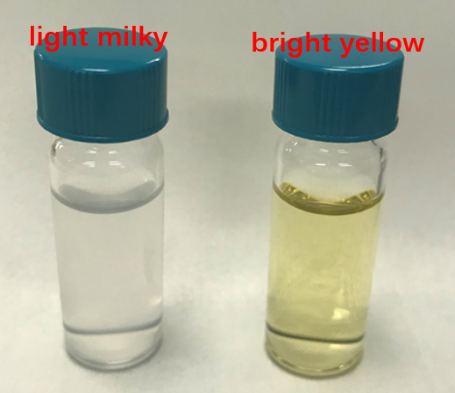
**

**Figure S2.** Digital images of SnO_2_ QDs solution. 2-AT additive assisted SnO_2_ QDs solution in the first stage (left) and the final stage (right) during the preparation process.


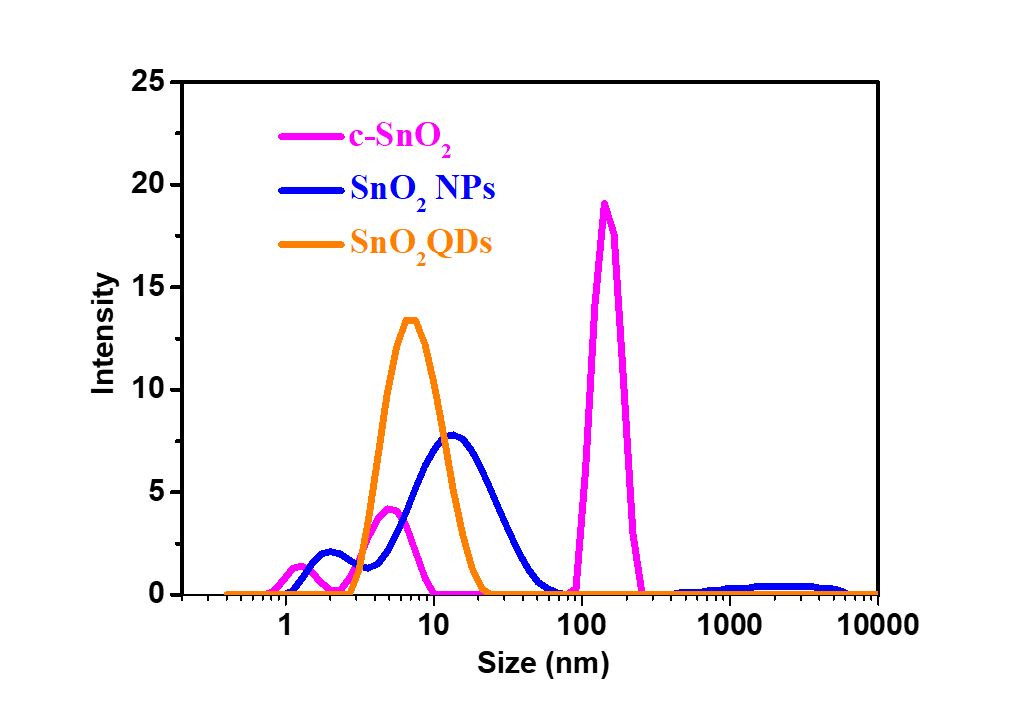


Figure S3. Particle size distribution via dynamic light scattering of c-SnO_2_ solution, SnO_2_ NPs solution and SnO_2_ QDs solution.

**
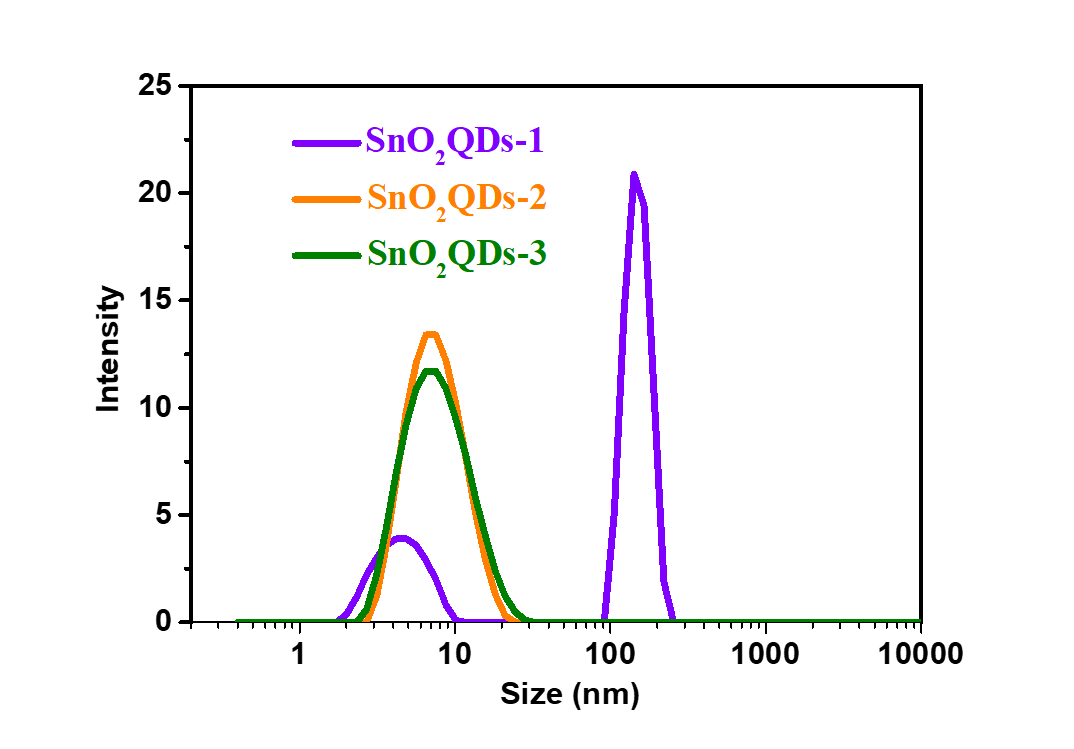
**

**Figure S4.** Particle size distribution via dynamic light scattering of SnO_2_ QDs solution by using different concentration of 2AT in the synthesis process. Note: SnO_2_ QDs-1 represent the decreased concentration of 2AT by 10 times during the synthesis process, SnO_2_ QDs-2 represent the original concentration of 2AT (see experimental section) during the synthesis process. SnO_2_ QDs-3 represent the increased concentration of 2AT by 5 times during the synthesis process.

**
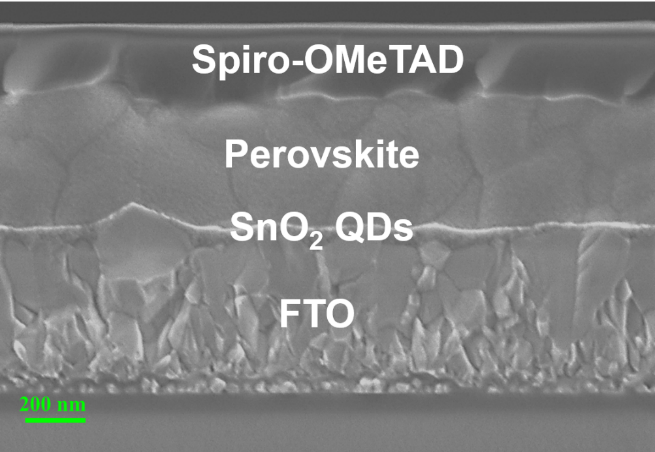
**

**Figure S5.** Cross-sectional SEM image and device architecture of SnO_2_ QDs based PSCs.

**
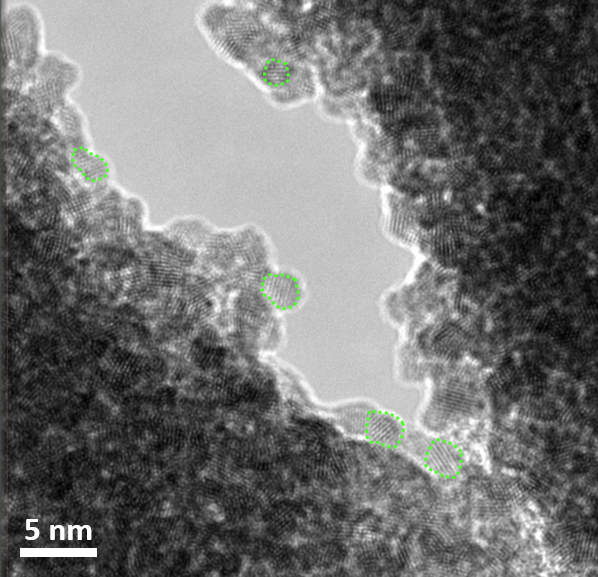
**

**Figure S6.** TEM image of SnO_2_ QDs coated on a carbon support copper grid.


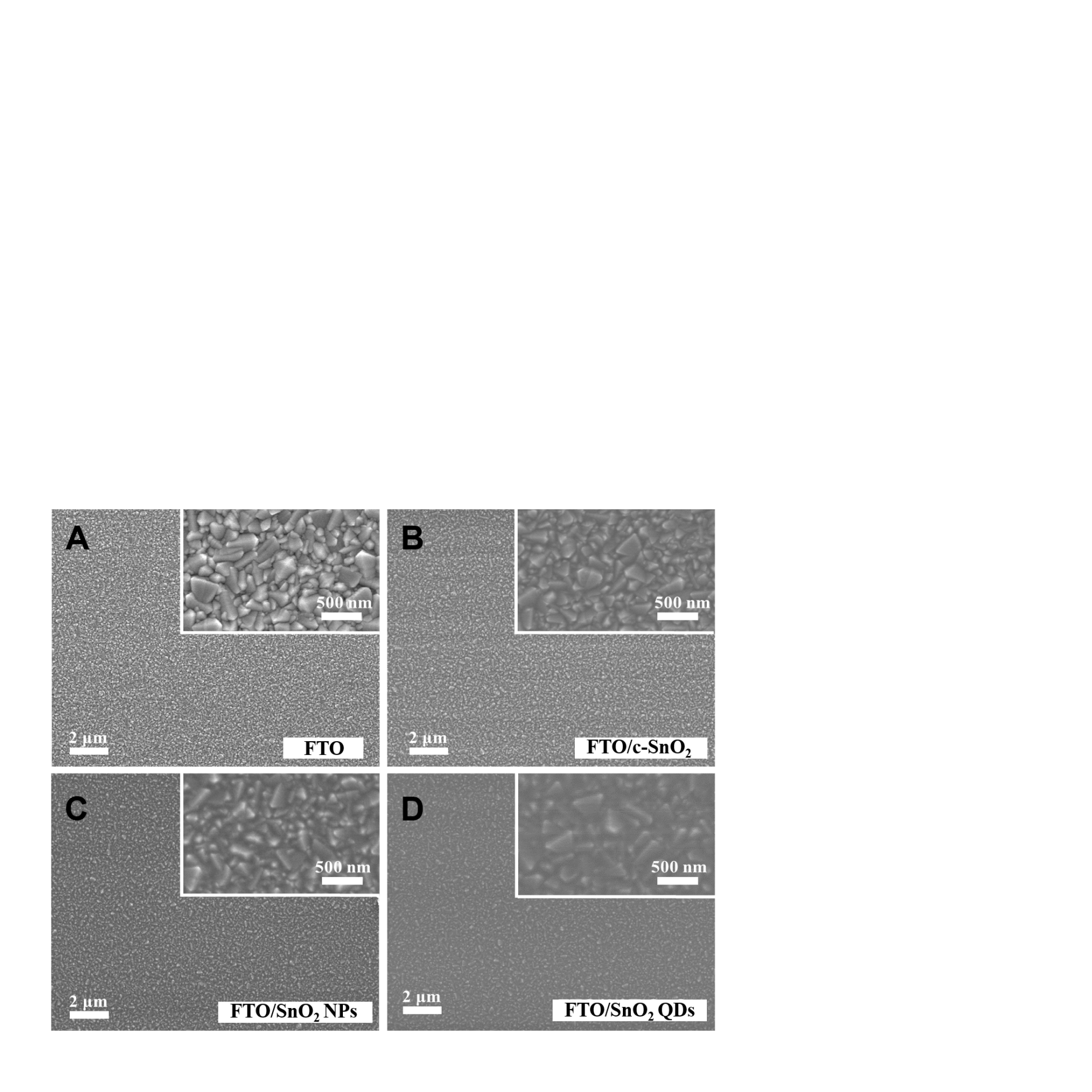


**Figure S7.** Top-view scanning electron microscopies of (A) bare FTO, (B) c-SnO_2_ coated FTO, (C) SnO_2_ NPs coated FTO and (D) SnO_2_ QDs coated FTO. The insets are the enlarged magnifications.


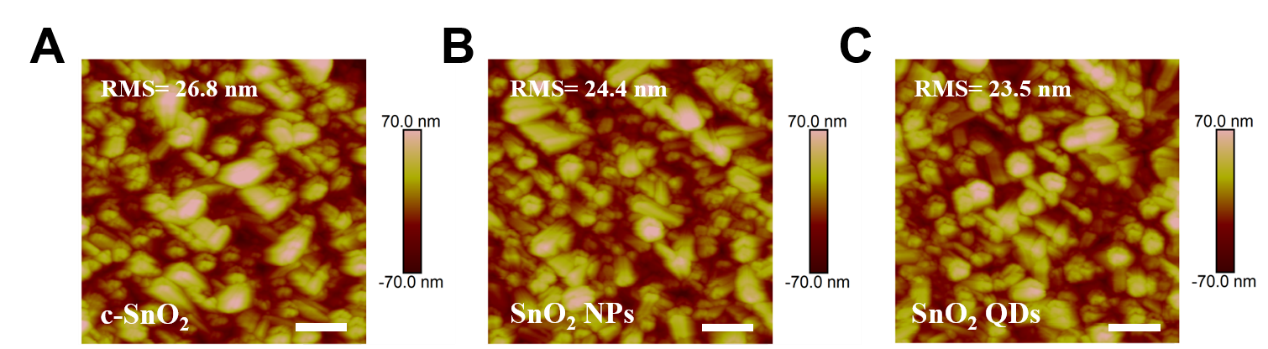


**Figure S8.** AFM images of (A) FTO/c-SnO_2_, (B) FTO/SnO_2_ NPs and (C) FTO/SnO_2_ NPs. Scale bar is 500 nm. SnO_2_ QDs sample show the smaller surface roughness (RMS: 23.5 nm) compared to c-SnO_2_ (RMS: 26.8 nm) and SnO_2_ NPs (RMS: 24.4 nm).

**
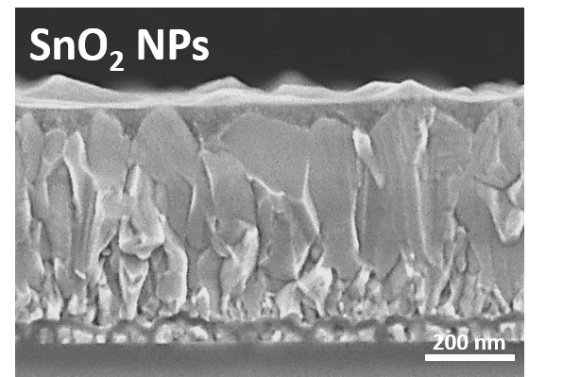
**

**Figure S9.** SEM cross-sectional image of SnO_2_ NPs coated FTO.


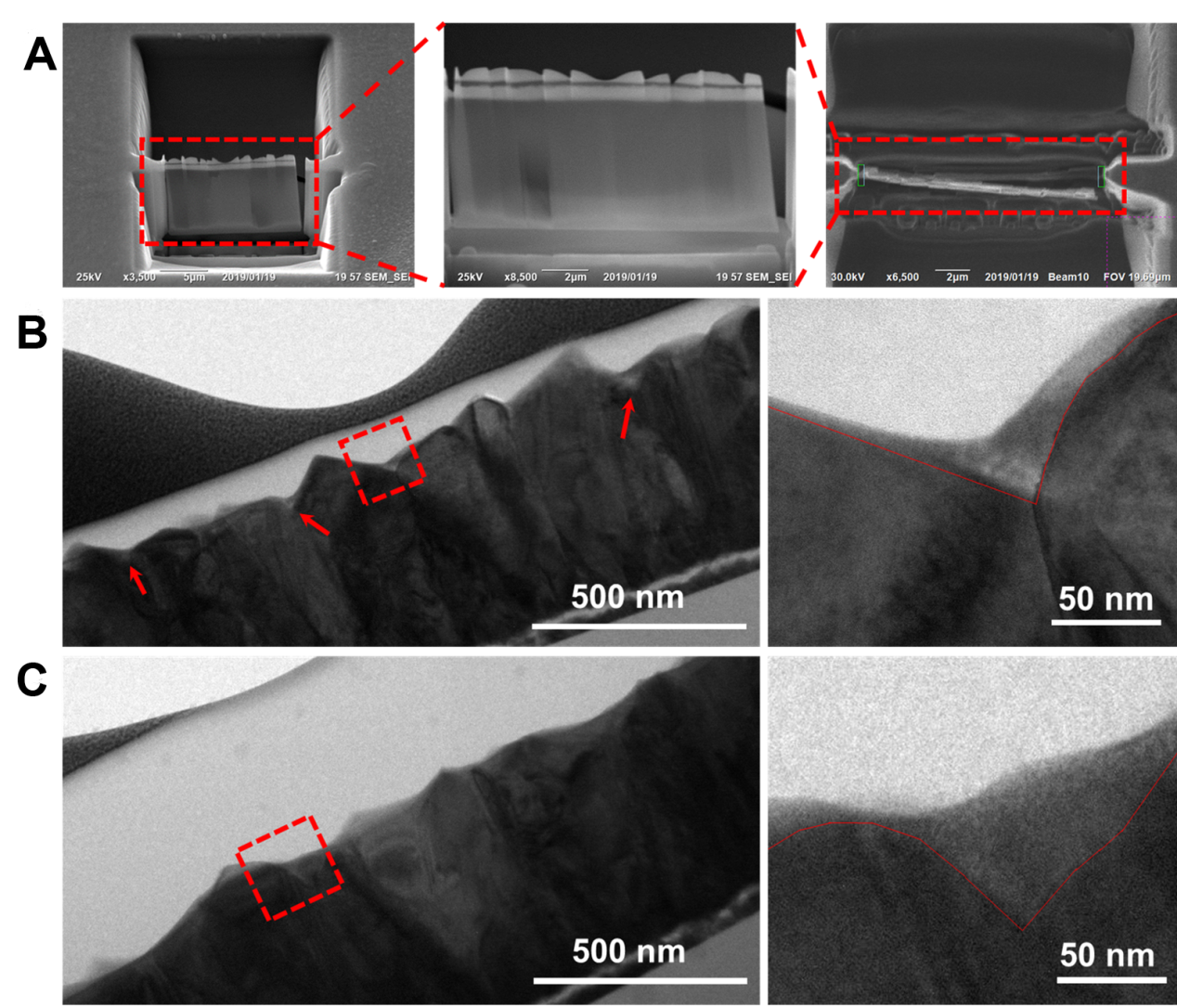


**Figure S10.** (A) SEM images (side view and top view) of S/TEM sample prepared by FIB technique. Cross-sectional TEM images of (B) c-SnO_2_/FTO and (C) SnO_2_ QDs/FTO. The arrows show the poor contact between the c-SnO_2_/FTO interface. Right images are the enlarged magnification version of the red dashed squares.

**
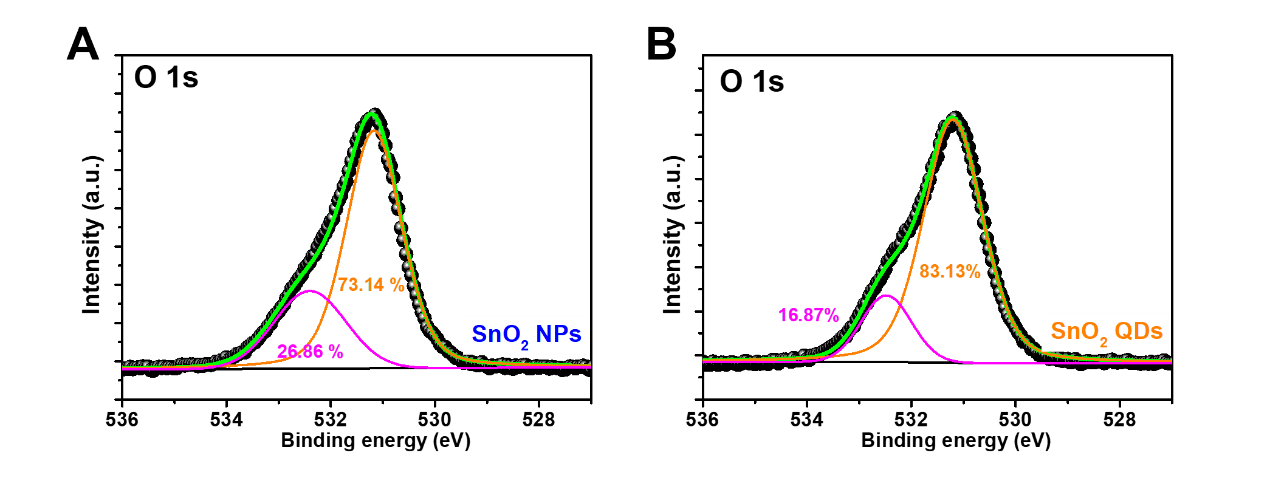
**

**Figure S11.** XPS core level spectra of O 1s in the (A) SnO_2_ NPs and (B) SnO_2_ QDs film.


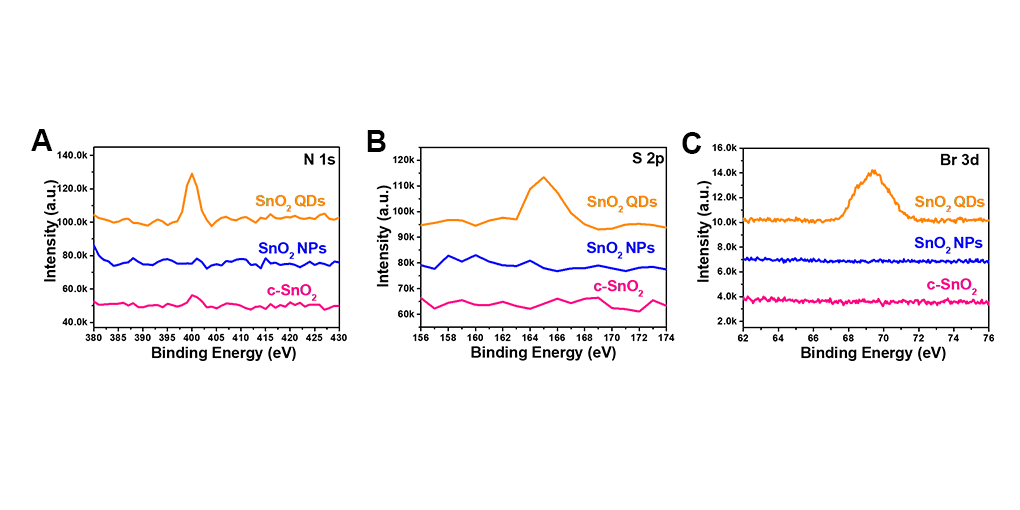


**Figure S12.** XPS core level spectra of (A) N 1s, (B) S 2p and (C) Br 3d on c-SnO_2_, SnO_2_ NPs and SnO_2_ QDs film.

**
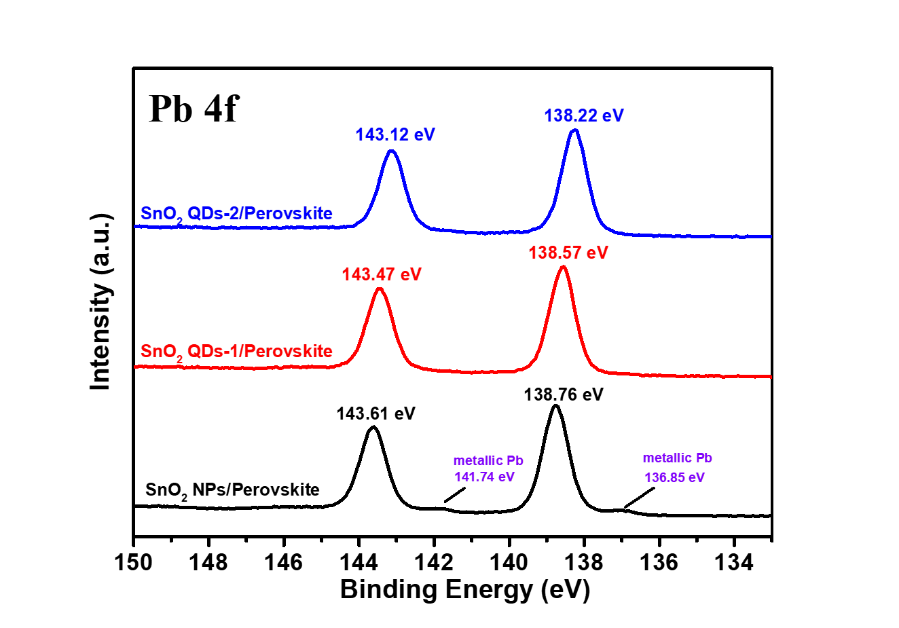
**

**Figure S13.** XPS core level spectra of Pb 4f in the perovskite films with SnO_2_ NPs and SnO_2_ QDs. Note: SnO_2_ QDs-1 represent the original concentration of 2AT (see experimental section) during the synthesis process, SnO_2_ QDs-2 represent the increased concentration of 2AT by 5 times during the synthesis process.


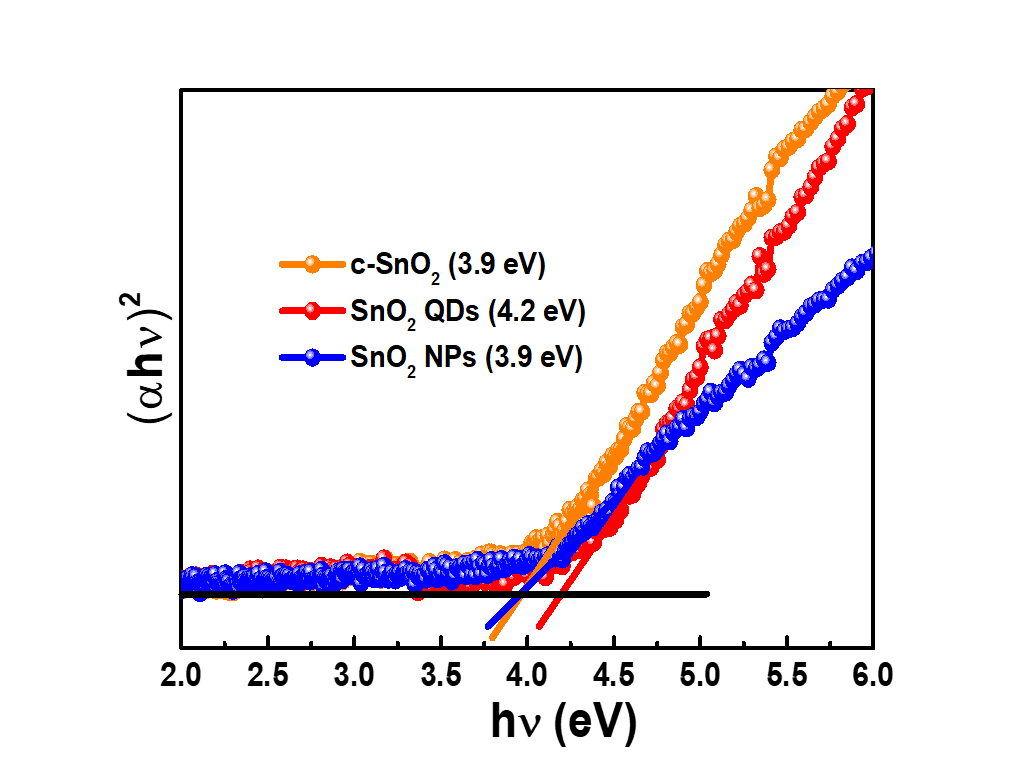


**Figure S14.** Tauc plot of c-SnO_2_, SnO_2_ NPs and SnO_2_ QDs films.


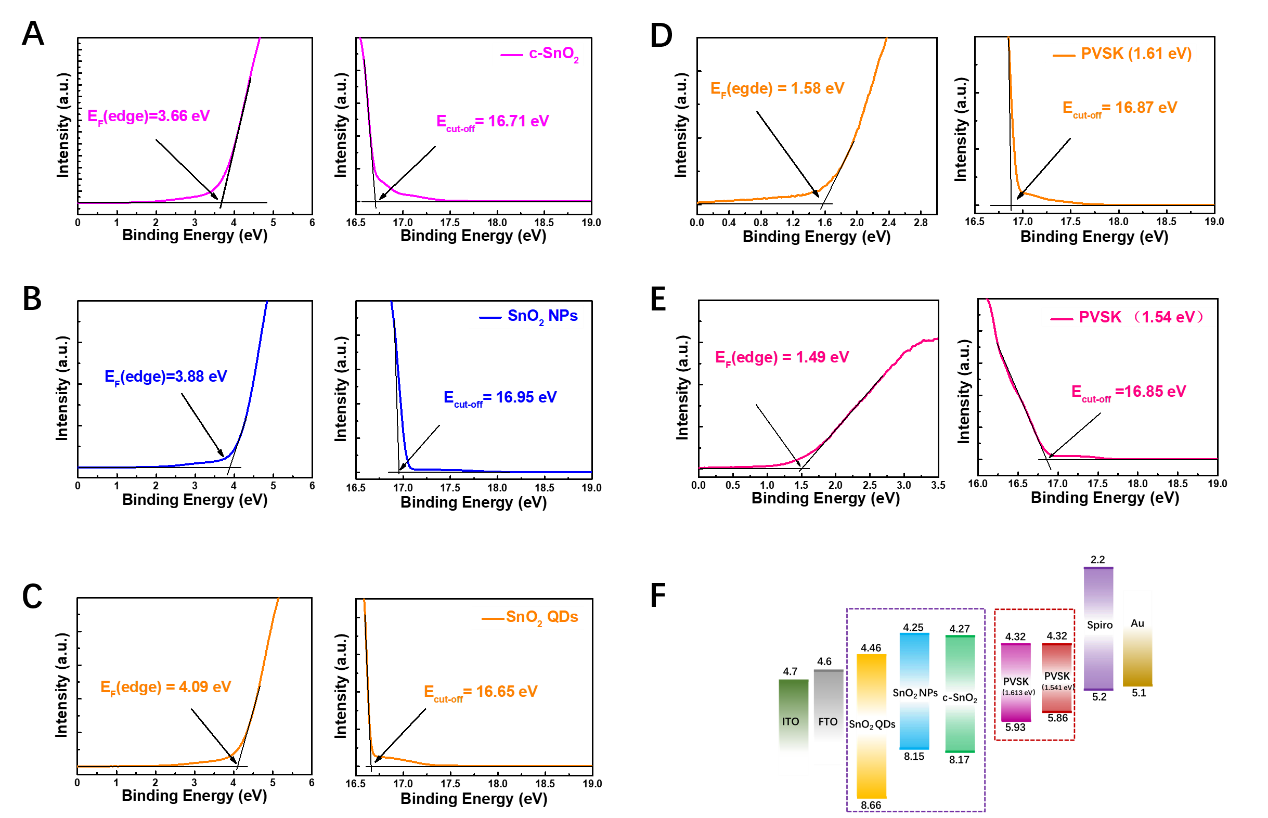


**Figure S15.** UPS spectra of (A) c-SnO_2_, (B) SnO_2_ NPs, (C) SnO_2_ QDs, perovskite with a bandgap of (D) 1.61 eV and (E) 1.54 eV. (F) Band energy level diagram of every single layer in our devices.

**Supplementary Note 1.**

As shown in Figure S15 (A-E), cut-off binding energy (E_cut-off_) is estimated to be 16.71 eV for c-SnO_2_, 16.95 eV for SnO_2_ NPs, 16.65 eV for SnO_2_ QDs, 16.87 eV for PVSK (1.61 eV) and 16.85 eV for PVSK (1.54 eV). According to the formula of E_F_=21.22 - E_cut-off_, Fermi level (E_F_) of c-SnO_2_, SnO_2_ NPs, SnO_2_ QDs, PVSK (1.61 eV) and PVSK (1.54 eV) were calculated to be -4.51 eV, -4.27 eV, -4.57 eV, -4.35 eV, and -4.37eV, respectively. Fermi edge (E_F_ (edge)) is -3.66 eV, -3.88 eV, -4.09 eV, -1.58eV, and -1.49 eV, for c-Sn for of c-SnO_2_, c-SnO_2_, SnO_2_ NPs, SnO_2_ QDs, PVSK (1.61 eV) and PVSK (1.54 eV). Based on the formula of E_VB_=E_F_- E_F_ (edge), it can be calculated that valence band maximum (E_VB_) of c-SnO_2_, SnO_2_ NPs, SnO_2_ QDs, PVSK (1.61 eV) and PVSK (1.54 eV) is -8.17 eV, -8.15 eV, -8.66 eV, -5.93 eV, and -5.86 eV, respectively. Furthermore, according to the equation of E_CB_=E_VB_ + E_g_, conduction band minimum (E_CB_) of c-SnO_2_, SnO_2_ NPs, SnO_2_ QDs, PVSK (1.61 eV) and PVSK (1.54 eV) was determined to be -4.27 eV, -4.25 eV, -4.46 eV, -4.32 eV, and -4.32 eV, respectively.


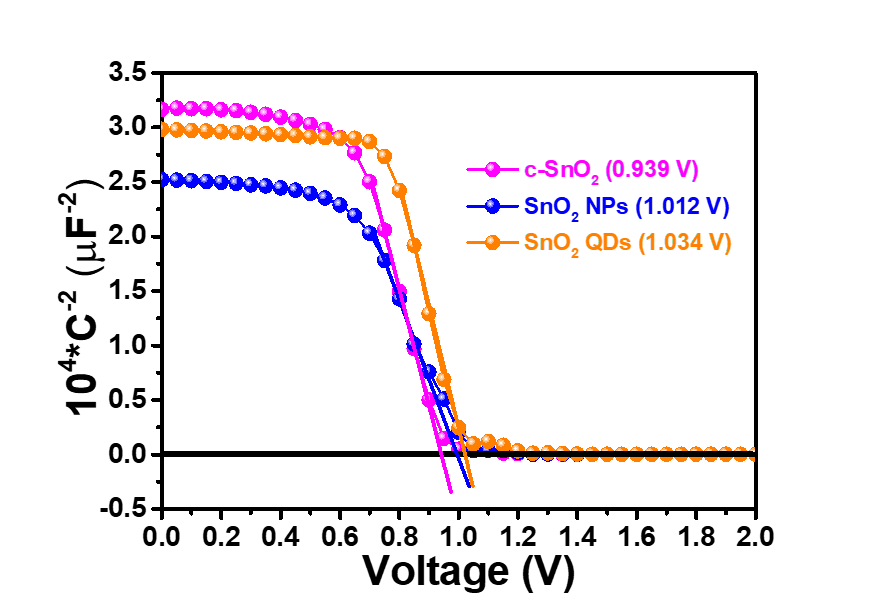


**Figure S16.** Mott-Schottky plots of PSCs with c-SnO_2_, SnO_2_ NPs and SnO_2_ QDs ETLs.

**Supplementary Note 2.**

Figure S9 displays the *V_bi_* of ETLs/Perovskite, which originating from the intercept of the linear area of Mott-Schottky plot with respect to the x-axis. We found that *V_bi_* value exhibits an increase from 0.939 V to 1.034 V by replacing c-SnO_2_ with SnO_2_ QDs as the ETL within the PSCs, while *V_bi_* value (1.012 V) of SnO_2_ NPs sample is lower than that of SnO_2_ QDs sample. The improvement in *V_bi_* observed in SnO_2_ QDs based PSCs is attributed to good interfacial physical contact and passivated interfacial contact.


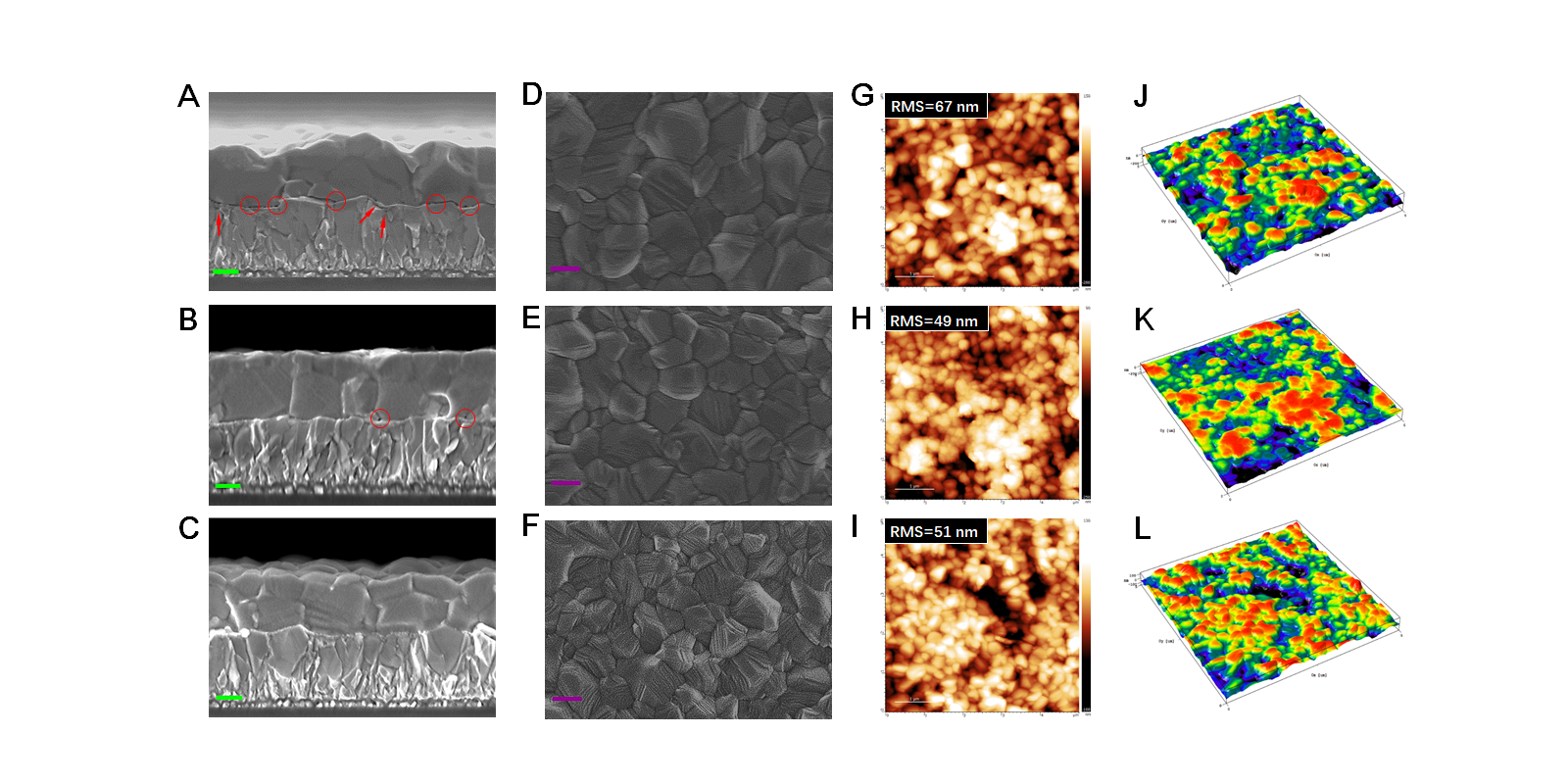


**Figure S17.** Cross-sectional SEM images of (A) FTO/c-SnO_2_/PVSK, (B) FTO/PVSK/SnO_2_ NPs and (C) FTO/PVSK/SnO_2_ QDs. Top-view SEM images of (D) FTO/c- SnO_2_/PVSK, (E) FTO/PVSK/SnO_2_ NPs and (F) FTO/PVSK/SnO_2_ QDs. Scale bar is 200 nm. The red circles highlight the poor physical contact between perovskite and ETLs (c-SnO_2_ and SnO_2_ NPs), showing that some area that the PVSK film indirect contact with ETLs (c-SnO_2_ and SnO_2_ NPs). The red arrows represent the pin holes between ETL (c-SnO_2_) and FTO layer. SnO_2_ QDs based sample demonstrate a smaller average crystal size compared to samples with c-SnO_2_ and SnO_2_ NPs ETLs. AFM images (5 µm-by-5 µm) of (G) FTO/c-SnO_2_/PVSK, (H) FTO/PVSK/SnO_2_ NPs and (I) FTO/PVSK/SnO_2_ QDs, and the corresponding 3D images (J to L). The roughness of samples with c-SnO_2_ ETLs (67 nm) is larger than that of SnO_2_ NPs based sample (49 nm) and SnO_2_ QDs based sample (51 nm).


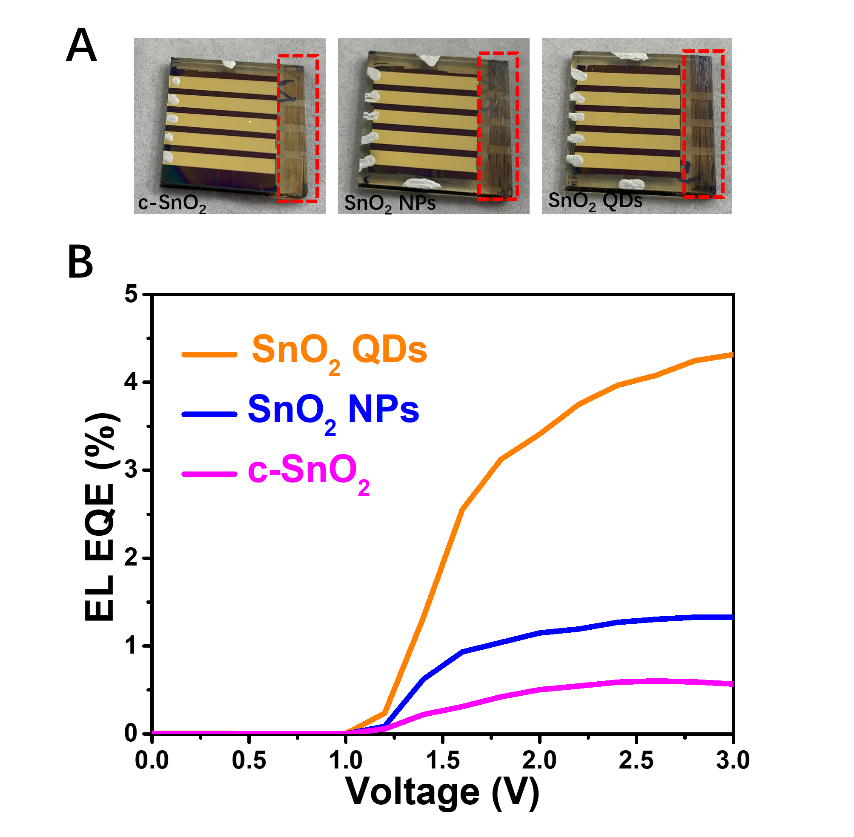


**Figure S18.** (**A**) Optical images of the real PSC devices with different ETLs. Red dashed rectangular show the scratched area. We observed that it can be easier to scratch the perovskite film off the c-SnO_2_/FTO substrate compared to SnO_2_ QDs (SnO_2_ NPs)/FTO substrates, which is an indicator of strengthened interfacial adhesion between perovskite and FTO substrates. (**B**) shows EL EQE as a function of voltage.


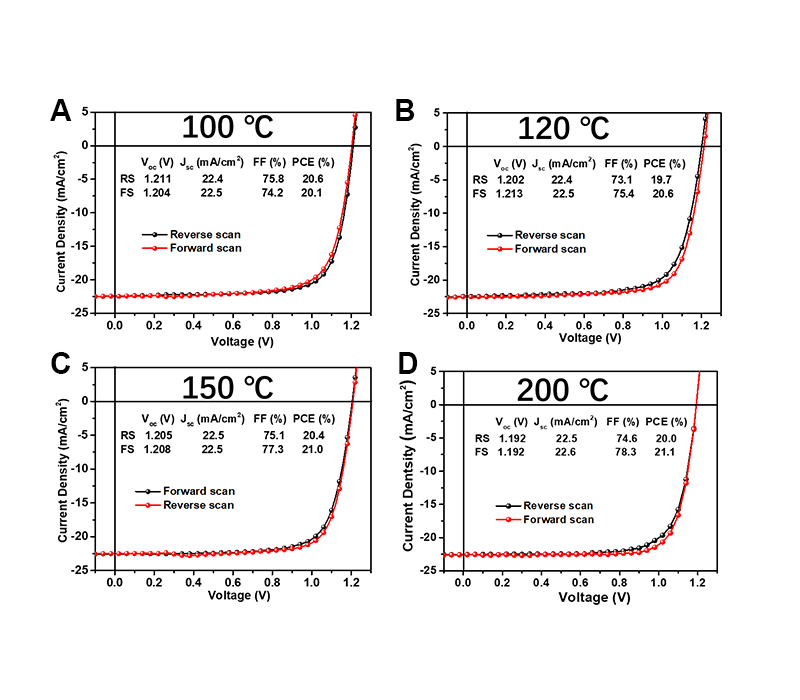


**Figure S19.** J-V curves of best-performing solar cells with different ETLs crystallization annealing temperature: (**A**) 100 ℃, (**B**) 120 ℃, (**C**) 150 ℃ and (**D**) 200 ℃. All devices were measured under forward and reverse scan directions.


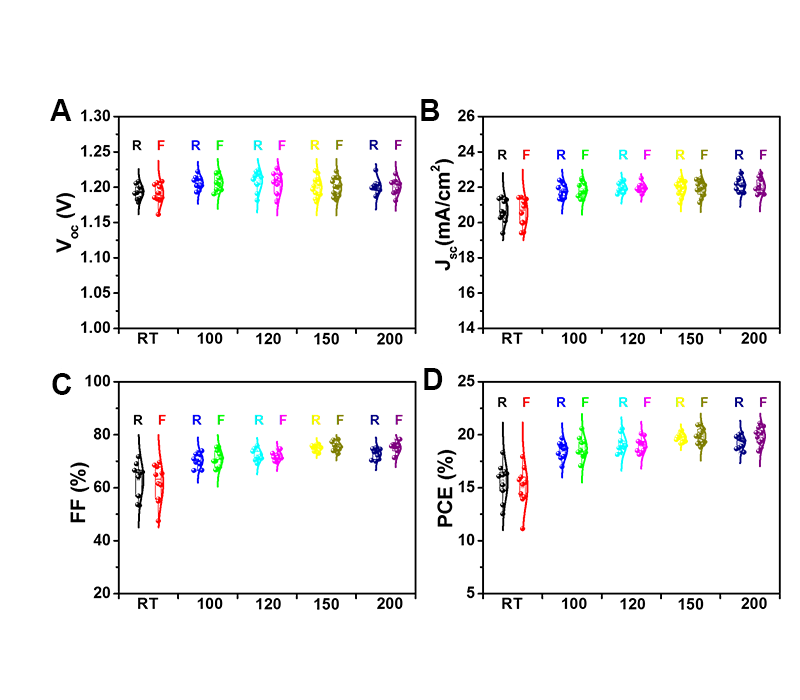


**Figure S20.** Histograms of the device photovoltaic parameters (**A**) *V_oc_*, (**B**) *J_sc_*, (**C**) FF, and (**D**) PCE obtained from a batch of PSCs with SnO_2_ QDs ETLs (different processing temperature). All the parameters (55 independent devices) were measured from forward and reverse scan directions.

**
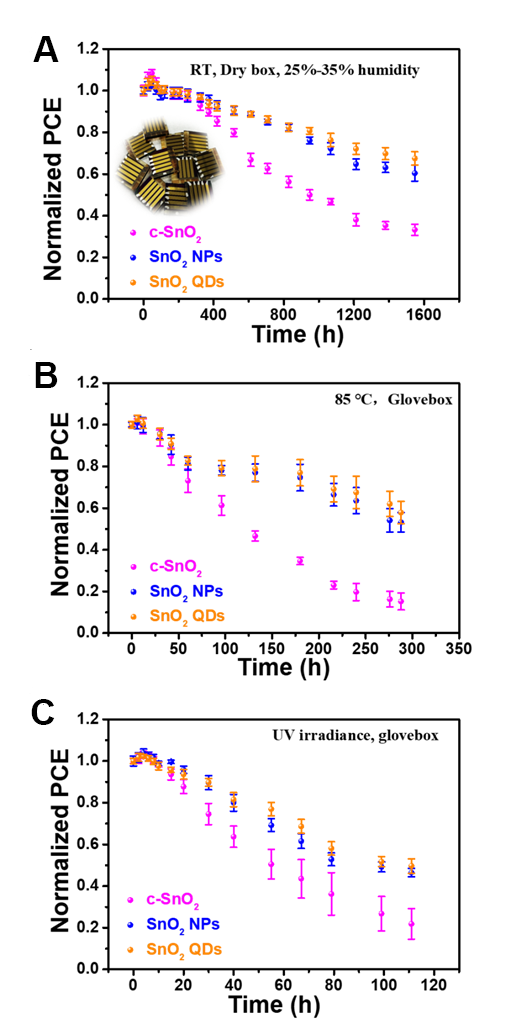
**

**Figure S21.** **Device stability.** (**A**) Dry box storage, (**B**) thermal aging test in glovebox, and (**C**) UV irradiation aging test in glovebox of the various ETLs based perovskite solar cells. PCE values in reverse scan condition were recorded in stability test. Note that the initial efficiencies of these pervoskite devices are about 18% for c-SnO_2_, 18% for SnO_2_ NPs and 19% for SnO_2_ QDs.


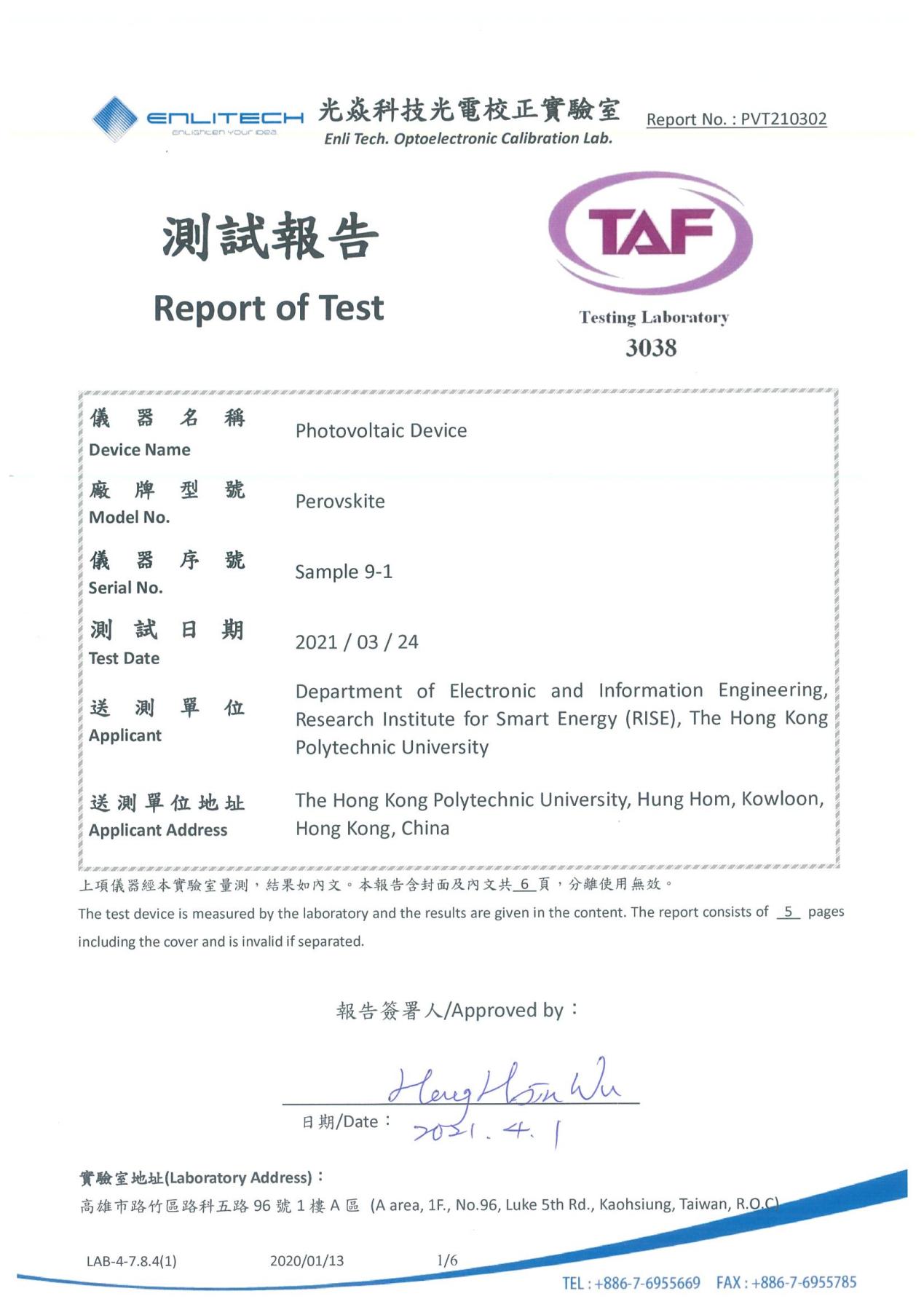


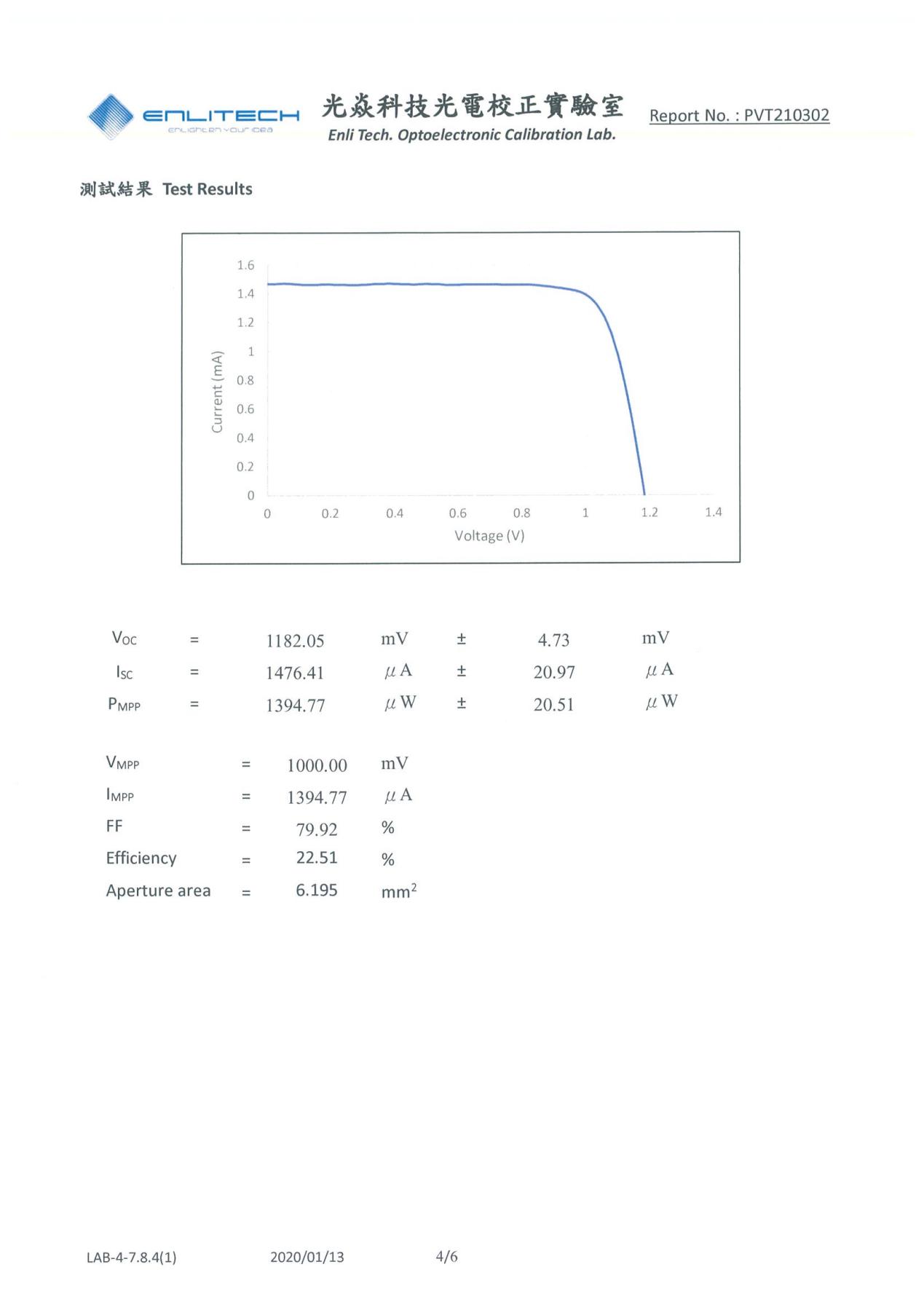


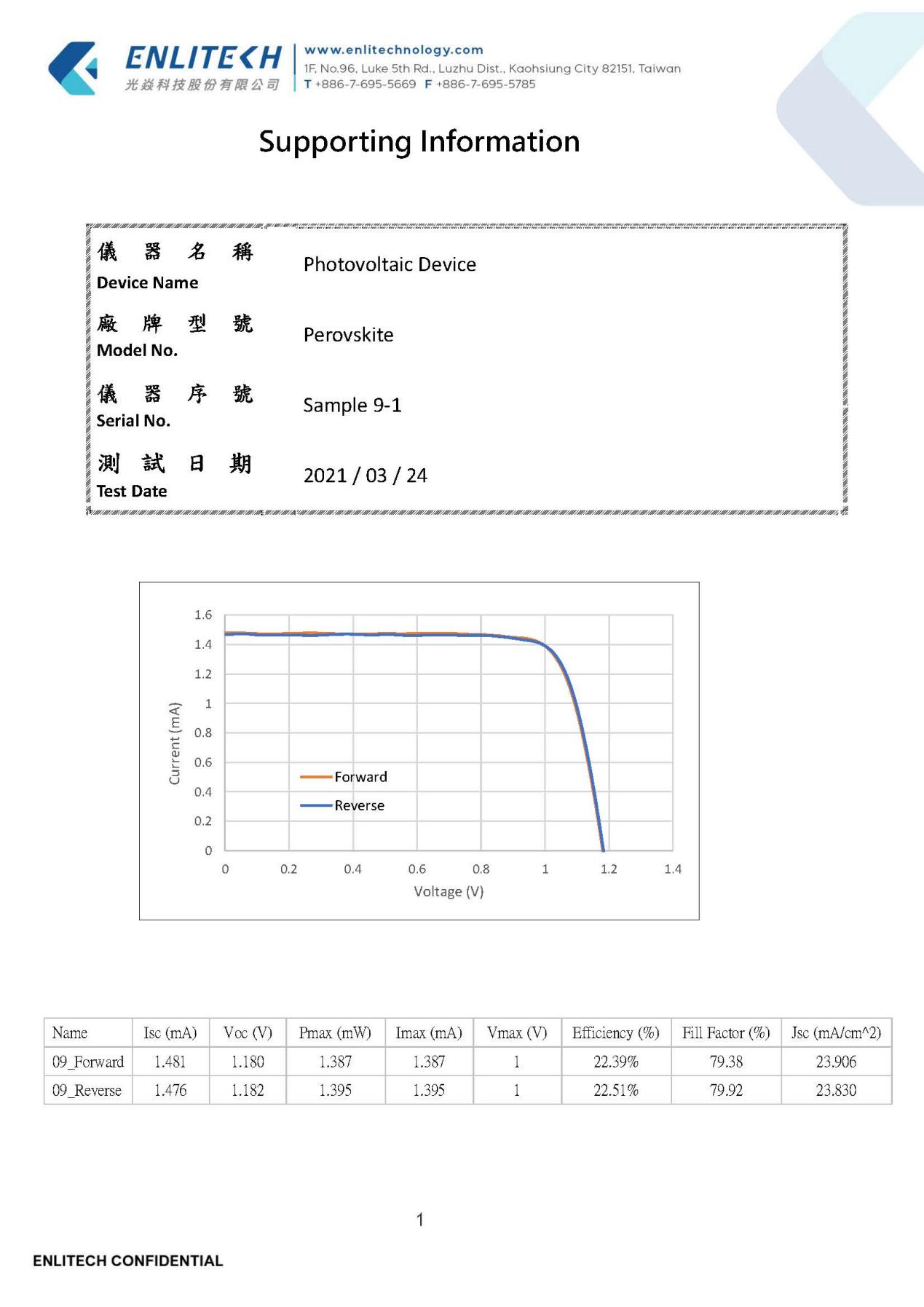


**Figure S22.** Certified results from an accredited photovoltaic certification laboratory (Enli Tech. Optoelectronic Calibration Lab.). The certified PCE is 22.51% - certified aperture with the area of 0.06195 cm^2^ (total active area 0.09 cm^2^). The certified J-V curves with double scanning give PCE_forward_: 22.39% and PCE_reverse_: 22.51%.


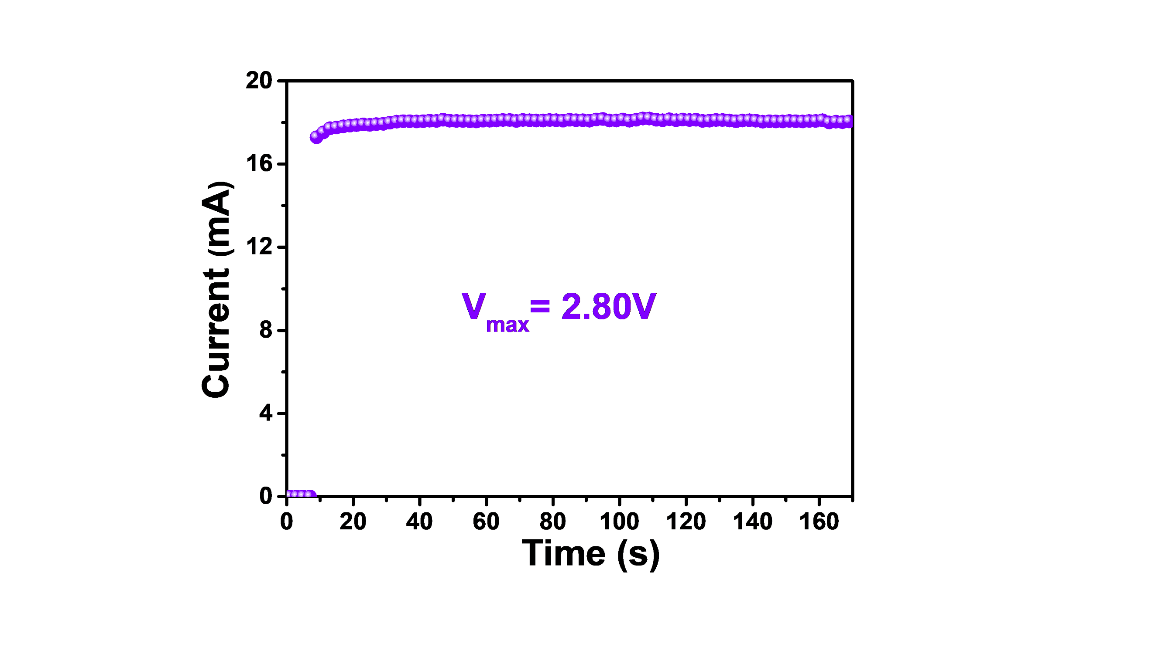


**Figure S23.** Steady-state current measurement of the mini module (1.613 eV, a total active area of 2.7 cm^2^) at the maximum voltage of 2.80 V, a stabilized PCE of 18.8%.


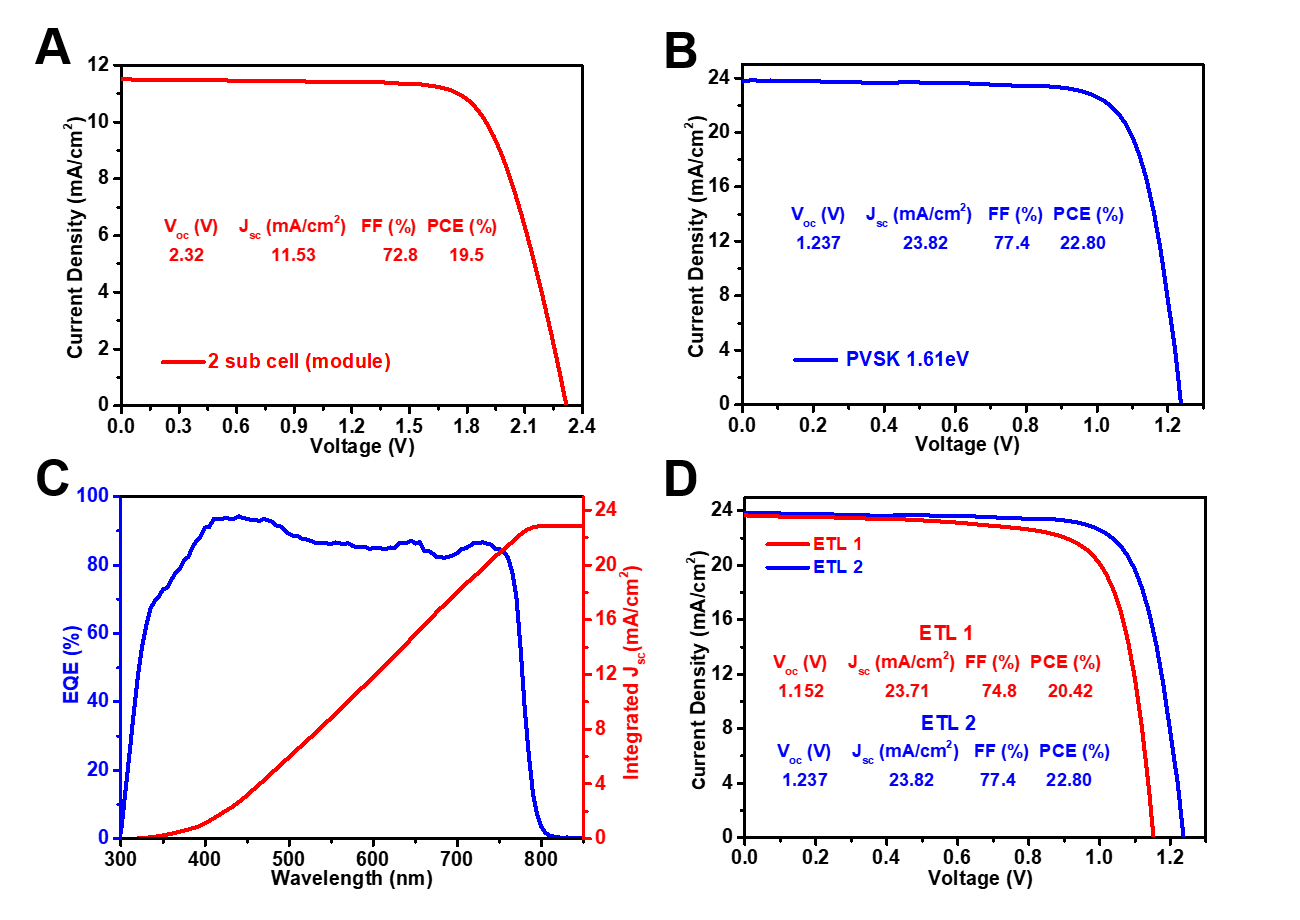


**Figure S24.** (**A**) J-V curves of a champion module with 1.54 eV perovskite system. (**B**) J-V curves, (**C**) EQE spectra and integrated current density of the best SnO_2_ QDs based perovskite solar cell with a bandgap of 1.61 eV, with ARC film. (**D**) J-V curves of the best performance blade-coated devices (1.61 eV) with ETL 1 and ETL 2. (ETL 1: commercial colloidal SnO_2_ NPs, ETL 2: multiple ligands-tailored SnO_2_ QDs)


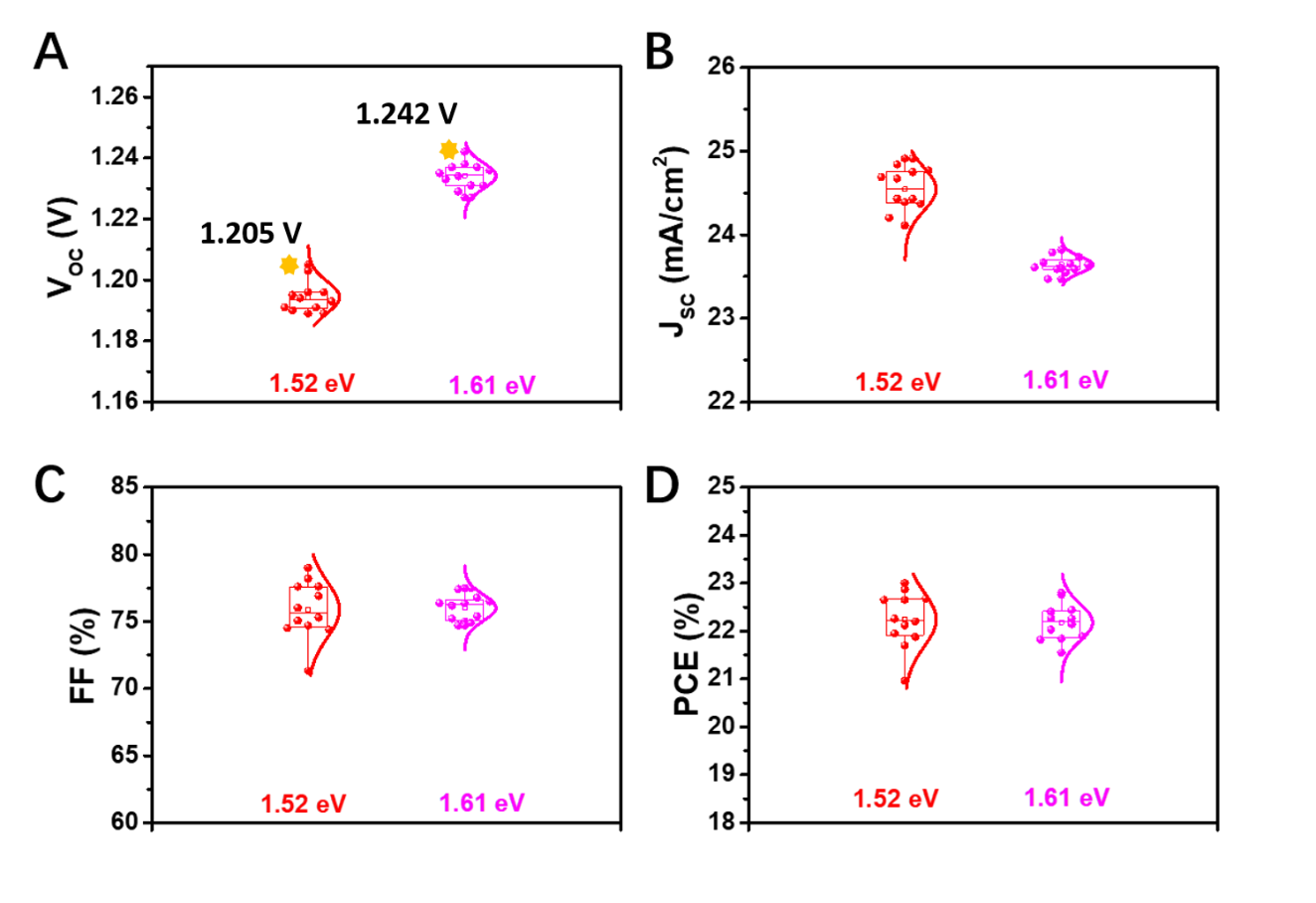


**Figure S25.** Histograms of the best-performing blade-coated PSCs with 1.54 eV and 1.61 eV bandgap. (**A**) *V_oc_*, (**B**) *J_sc_*, (**C**) FF, and (**D**) PCE. The yellow seven stars represent the highest *V_OC_* of PSCs with 1.54 eV and 1.61 eV bandgap.

**
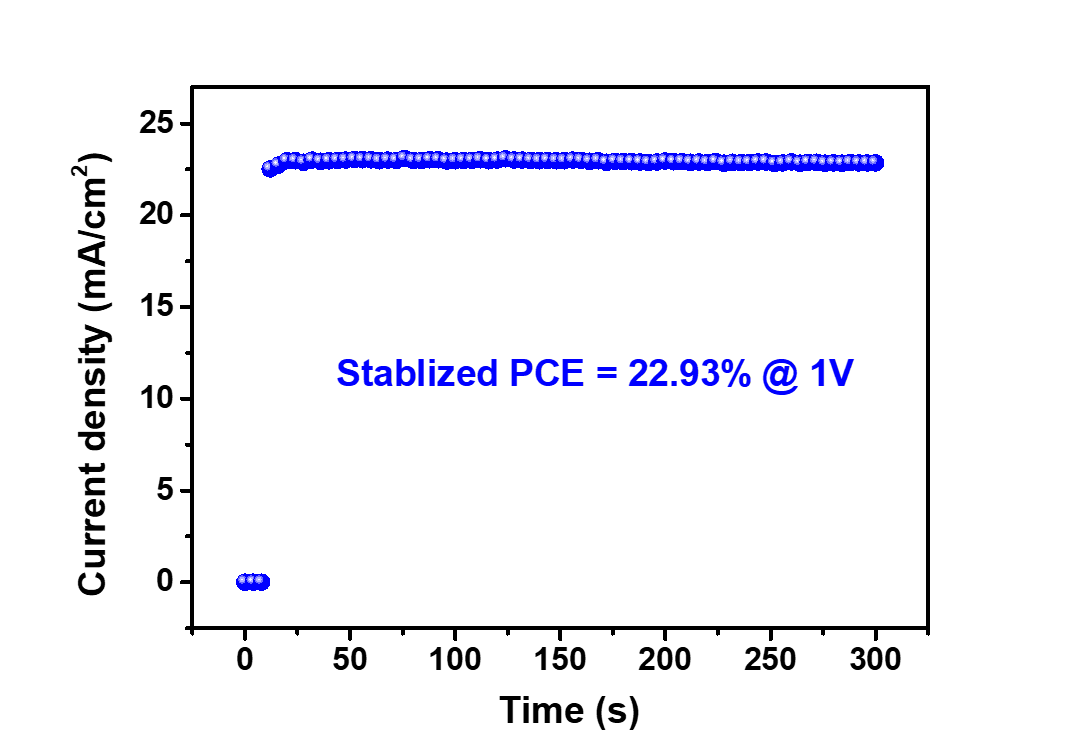
**

**Figure S26.** Steady-state output at the maximum power point of (*V_max_* of 1.00 V) of the best-performing blade-coated PSCs with 1.54 eV bandgap.


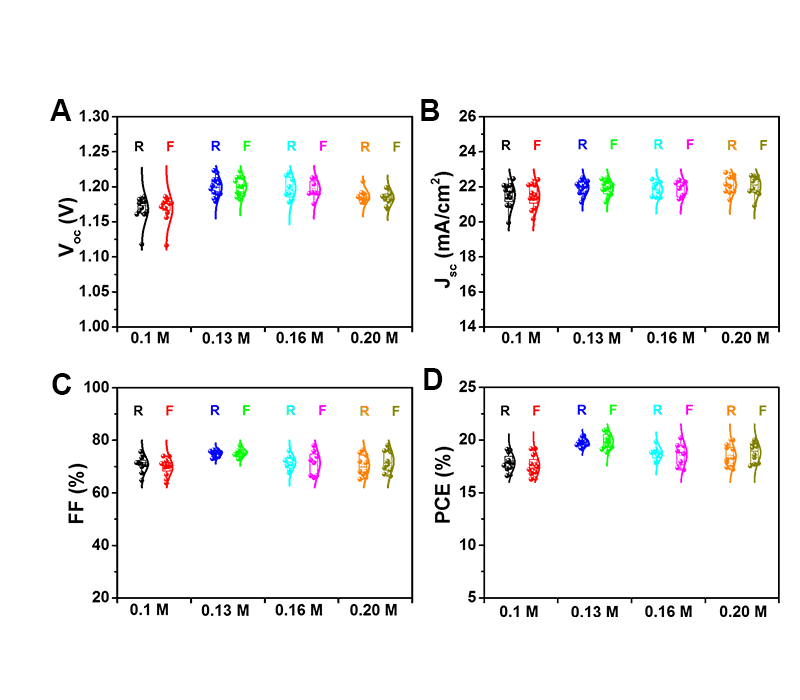


**Figure S27.** Histograms of the device photovoltaic parameters (**A**) *V_oc_*, (**B**) *J_sc_*, (**C**) FF, and (**D**) PCE obtained from a batch of PSCs with SnO_2_ QDs ETLs (different concentrations: 0.1 M, 0.13 M, 0.16 M and 0.2 M). All the parameters (38 independent devices) were measured from forward and reverse scan directions.

Table S1. The band parameters of c-SnO_2_, SnO_2_ NPs, SnO_2_ QDs and perovskite.

| Sample | E_g_  (eV) | E_F_ (edge)  (eV) | E_F_ (cut-off)  (eV) | W_F_  (eV) | E_VB_  (eV) | E_CB_  (eV) |
| --- | --- | --- | --- | --- | --- | --- |
| c-SnO_2_ | 3.9 | 3.66 | 16.71 | -4.51 | -8.17 | -4.27 |
| SnO_2_ NPs | 3.9 | 3.88 | 16.95 | -4.27 | -8.15 | -4.25 |
| SnO_2_ QDs | 4.2 | 4.09 | 16.66 | -4.57 | -8.66 | -4.46 |
| Perovskite (1.61eV) | 1.61 | 1.58 | 16.87 | -4.35 | -5.93 | -4.32 |
| Perovskite (1.54 eV) | 1.54 | 1.49 | 16.85 | -4.37 | -5.86 | -4.32 |

Table S2. The EL parameters of typical device with c-SnO_2_, SnO_2_ NPs and SnO_2_ QDs.

| Sample | **∆** $\boldsymbol{V}_{\boldsymbol{oc}}^{non\boldsymbol{rad}}$ | EL EQE_Jsc injection_ (%) | EL EQE_maximum_ (%) | *V_oc_* (V) |
| --- | --- | --- | --- | --- |
| c-SnO_2_ | 0.187 | 0.08 | 0.6 | 1.158 |
| SnO_2_ NPs | 0.162 | 0.12 | 1.3 | 1.168 |
| SnO_2_ QDs | 0.133 | 0.61 | 4.4 | 1.202 |

Table S3. The PV parameters of the best-performing c-SnO_2_, SnO_2_ NPs and SnO_2_ QDs based CB-antisolvent process PSCs (1.61 eV) obtained by both FS (-0.2 to 1.2 V) and RS (1.2 to -0.2 V) directions.

| Device | | *V_oc_* (V) | *J_sc_* (mA cm^-2^) | FF (%) | PCE (%) | SPO (%) | \|h-index\| |
| --- | --- | --- | --- | --- | --- | --- | --- |
| c-SnO_2_ | R | 1.161 | 21.7 | 76.7 | 19.3 | 18.8 | 0.09 |
|  | F | 1.148 | 21.6 | 70.8 | 17.6 |  |  |
| SnO_2_ NPs | R | 1.159 | 22.5 | 75.6 | 19.8 | 19.7 | 0.03 |
|  | F | 1.156 | 22.5 | 74.3 | 19.3 |  |  |
| SnO_2_ QDs | R | 1.205 | 22.5 | 75.1 | 20.4 | 21.2 | 0.03 |
|  | F | 1.208 | 22.5 | 77.3 | 21.0 |  |  |

Table S4. The PV parameters of the best-performing blade-coated PSCs with different active area.

| Perovskite  Bandgap (eV)^a^ | Active area  (cm^2^) | *V_oc_* (V)  (*V_oc_* max) | | *V_oc_* loss (V) | *J_sc_* (*J_sc_* cal.)  (mA cm^-2^)  (With ARC film) | FF (%) | PCE (%) |
| --- | --- | --- | --- | --- | --- | --- | --- |
| 1.541 | 0.04 | | 1.189  (1.201) | 0.340 | 24.75  (23.90) | 78.2 | 23.02 |
|  | 0.98 | | 1.197  (1.205) | 0.336 | 24.48 | 73.8 | 21.62 |
|  | 1.8  (2-subcells) | | 2.32 | - | 11.53 | 72.8 | 19.47 |
| 1.613 | 0.04 | | 1.237  (1.242) | 0.371 | 23.82 | 77.4 | 22.80 |
|  | 0.8 | | 1.186 | - | 23.52 | 74.2 | 20.70 |
|  | 2.7  (3-subcells) | | 3.51 | - | 7.54 | 71.3 | 18.87 |

^a^Perovskite bandgap was derived from the average value of *E_g_^IP^*, which can be calculated by the maximum differential value of EQE.
